# The MTR4 helicase recruits nuclear adaptors of the human RNA exosome using distinct arch-interacting motifs

**DOI:** 10.1038/s41467-019-11339-x

**Published:** 2019-07-29

**Authors:** Mahesh Lingaraju, Dennis Johnsen, Andreas Schlundt, Lukas M. Langer, Jérôme Basquin, Michael Sattler, Torben Heick Jensen, Sebastian Falk, Elena Conti

**Affiliations:** 1 Department of Structural Cell Biology, Max-Planck-Institute of Biochemistry, Am Klopferspitz 18, D-82152 Martinsried, Germany; 2 Department of Molecular Biology and Genetics, Aarhus University, C.F. Møllers Alle 3, 8000 Aarhus C, Denmark; 3 Center for Integrated Protein Science Munich (CIPSM) at Department of Chemistry, Technical University of Munich (TUM), 85747 Garching, Germany; 4 Institute of Structural Biology, Helmholtz-Zentrum München, 85764 Neuherberg, Germany; 5 Present Address: Institute for Molecular Biosciences and Center for Biomolecular Magnetic Resonance (BMRZ) at Johann Wolfgang Goethe-University, Frankfurt am Main, 60438 Germany; 6 Present Address: Max F. Perutz Laboratories, Department of Structural and Computational Biology, University of Vienna, Campus Vienna Biocenter 5, 1030 Vienna, Austria

**Keywords:** NMR spectroscopy, X-ray crystallography, RNA, RNA decay

## Abstract

The nuclear exosome and its essential co-factor, the RNA helicase MTR4, play crucial roles in several RNA degradation pathways. Besides unwinding RNA substrates for exosome-mediated degradation, MTR4 associates with RNA-binding proteins that function as adaptors in different RNA processing and decay pathways. Here, we identify and characterize the interactions of human MTR4 with a ribosome processing adaptor, NVL, and with ZCCHC8, an adaptor involved in the decay of small nuclear RNAs. We show that the unstructured regions of NVL and ZCCHC8 contain short linear motifs that bind the MTR4 arch domain in a mutually exclusive manner. These short sequences diverged from the arch-interacting motif (AIM) of yeast rRNA processing factors. Our results suggest that nuclear exosome adaptors have evolved canonical and non-canonical AIM sequences to target human MTR4 and demonstrate the versatility and specificity with which the MTR4 arch domain can recruit a repertoire of different RNA-binding proteins.

## Abstract (web summary)

The human RNA exosome contains a nuclear co-factor MTR4, which unwinds structural RNAs and recruits adaptors for different RNA processing and decay pathways. Here the authors uncover new variations of the arch-interacting motif (AIM) in NVL and ZCCHC8 and characterise their interaction with MTR4.

## Introduction

Eukaryotic cells generate a multitude of RNA species that require timely maturation and decay to maintain a healthy transcriptome. A central machinery in nuclear RNA processing, quality control and decay pathways is a conserved 3′–5′ exoribonuclease complex known as the RNA exosome (reviewed in^1,2^). Most mechanistic studies to date have analyzed the RNA exosome from *S. cerevisiae*, the species in which it was originally identified 20 years ago^3^. The yeast exosome consists of a 10-subunit core complex (Exo_10_)^4^, the activity of which depends on a single processive exoribonuclease (Rrp44, also known as Dis3)^5,6^. The Exo_10_ core is present in both nuclear and cytoplasmic compartments, but its cofactors and regulators have distinct subcellular localizations (reviewed in^1,2^). In the nucleus, Exo_10_ is bound to the distributive ribonuclease Rrp6 and its associated protein Rrp47 as well as to the Mpp6 protein^7–9^. Together, Rrp6-Rrp47 and Mpp6 recruit the RNA helicase Mtr4 to the exosome^10–12^. Orthologues of all these 14 proteins exist in human cells, and engage in similar interactions to form the corresponding human nuclear exosome complex^12,13^.

In both yeast and humans, the nuclear helicase Mtr4/MTR4 is central to exosome function^2,14,15^. First, it functions as an enzyme to remodel ribonucleoprotein (RNP) substrates with its 3′–5′ unwinding activity, and to present the unwound RNA substrate to the exosome core^12,16^. Furthermore, it functions as a binding platform for RNA-binding adaptors, providing the primary interactions to transcripts subjected to exosomal degradation in both RNA processing and decay pathways^17,18^. For example, *S. cerevisiae* Mtr4 binds Nop53, a ribosome biogenesis factor that recruits the exosome for a late step in rRNA processing, namely the trimming of ITS2 (Internal Transcribed Spacer 2). Two other factors, Trf4 and Air2, bind Mtr4 to form the so-called TRAMP complex^19–21^, which allows the exosome to target aberrant tRNAs^22^, rRNAs and small nuclear and nucleolar RNAs (sn/snoRNAs) for decay^23^. Higher eukaryotes not only have orthologues of Nop53 and TRAMP, but also have an increased number of nuclear exosome adaptors. In human cells, MTR4 has been reported to interact with the early ribosome biogenesis factors WDR74 and NVL, which take part in the processing of ITS1 (Internal Transcribed Spacer 1)^24,25^. Human MTR4 also binds to two large Zinc-finger proteins, ZCCHC8 and ZFC3H1. ZCCHC8 interacts with MTR4 and the RNA-binding protein RBM7 to form the trimeric Nuclear EXosome Targeting (NEXT) complex^17,26^, which targets enhancer RNAs (eRNAs), promoter upstream transcripts (PROMPTs) and intronic RNAs for exosome-mediated decay^27^. ZFC3H1 instead directs MTR4 and the nuclear exosome to polyadenylated nuclear RNAs by connecting to the nuclear poly(A) binding protein PABN1^28–30^. Furthermore, MTR4 binds NRDE-2, a negative regulator that prevents the nuclear exosome  from targeting RNAs that should be exported to the cytoplasm^31^.

How does the nuclear exosome helicase mediate binding to such a diverse and functionally distinct set of proteins? Structural studies have shown that yeast Mtr4 contains an unstructured N-terminal region, a DExH helicase core and an ‘arch’ domain with a globular Kyprides, Ouzounis, Woese (KOW) domain^14,32^. All Mtr4 domains are involved in protein-protein interactions: the N-terminal region binds Rrp6-Rrp47^11^, the helicase core binds Mpp6 as well as Trf4-Air2^33,34^ and the KOW domain binds a short sequence known as ‘arch-interacting motif’ that is present in Nop53, Utp18, and Air2^35,36^. In the case of human MTR4, both the arch domain and the DExH helicase core bind NRDE-2^31^. Structural data have also elucidated how the helicase core of human MTR4 binds a region of ZCCHC8^37^. This protein is however expected to harbor another MTR4-binding region^37^, which remains to be identified. Also unclear is how other human MTR4-binding proteins are recognized. With the exception of the expected AIM sequence in the human NOP53 orthologue (also known as GLTSCR2) and in NRDE-2^31^, there is no consensus motif that can be identified with confidence at the sequence level in other MTR4-binding factors. Here, we used a combination of biochemical studies, X-ray crystallography and nuclear magnetic resonance (NMR) experiments to obtain mechanistic insights into how human MTR4 interacts with two metazoan RNA exosome adaptors: the RNA processing factor NVL and the RNA decay factor ZCCHC8.

## Results

### The unstructured region of human NVL interacts with MTR4

**Fig. 1:**
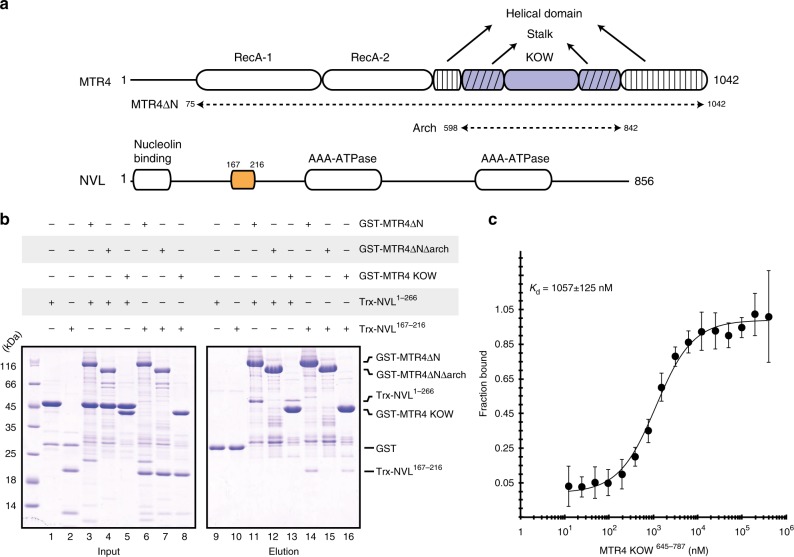
The N-terminal unstructured region of NVL interacts with the MTR4 KOW. **a** Schematic representation of the domain organization of NVL and MTR4. Domain boundaries are obtained from previous studies^31,37^ and the crystal structure in the current report. The arrows indicate nomenclature for MTR4 constructs used in the text. **b** Protein co-precipitation assays. GST-tagged MTR4∆N, MTR4∆N∆arch, and MTR4 KOW were incubated with either Trx-NVL^1–266^ or Trx-NVL^167–216^ before co-precipitation with glutathione sepharose beads. A total of 3% of the input (lanes 1–8) and 30% of the eluates (lanes 9–16) were analyzed on 15% SDS-PAGE gels stained with coomassie brilliant blue. **c** Microscale thermophoresis experiment with MTR4 KOW and Trx-NVL^167–216^-(GS)_3_-eYFP. In all, 50 nM of Trx-NVL^167–216^-(GS)_3_-eYFP was incubated with increasing concentrations of MTR4 KOW and thermophoresis was measured by tracking the fluorescence of the NVL-YFP fusion protein. The binding isotherm was calculated using MO software (Nanotemper technologies) and the dissociation constant (*K*_d_) is given in the inset. The error bars represent the standard deviations of each data point calculated from three independent thermophoresis measurements

The nuclear VCP-like (NVL) protein, also known as NVL2, is a ribosome biogenesis factor of the AAA-ATPase family that has been reported to interact with human MTR4^38^. NVL is a multidomain protein characterized by an N-terminal nucleolin-binding domain (residues 10–74)^39^ and a linker region (residues 76–266) followed by two globular domains, characteristic of AAA-ATPases that are responsible for catalytic activity (Fig. 1a, Supplementary Fig. 1a). Bioinformatic analyses suggested that the linker region is mostly unstructured (residues 76–239) (Supplementary Fig. 1b). In other AAA-ATPases, unstructured regions upstream of the catalytic domains often mediate protein-protein interactions^40–42^. To test if the portion upstream of the NVL catalytic domains mediates the interaction with MTR4, we expressed and purified the N-terminal region of human NVL tagged with thioredoxin (Trx) as the prey protein (Trx-NVL^1–266^) and the structured portion of MTR4 tagged with glutathione-S-transferase (GST) as the bait protein (GST-MTR4-∆N). In these assays, Trx-NVL^1–266^ specifically co-precipitated with GST-MTR4-∆N (Fig. 1b, lane 11, compare with GST control in lane 9). Interestingly, pull-down assays with the recombinant yeast orthologues showed that the N-terminal unstructured region of Rix7 does not interact with yeast Mtr4 (Supplementary Fig. 1c), indicating that the interaction between human NVL and MTR4 is not conserved in *S. cerevisiae*.

We narrowed down the MTR4-interacting region of human NVL based on bioinformatic analysis. Sequence alignments showed that the N-terminal unstructured region of NVL contains an insertion that is present in the human protein and other chordates but not in the yeast orthologue (Supplementary Fig. 1a). Upon testing whether this insertion is responsible for the interaction with human MTR4, we found that a construct encompassing the conserved portion of the human NVL insertion spanning residues 167–216 (Supplementary Fig. 1a) was indeed able to co-precipitate with GST-MTR4-∆N in pull-down assays (Fig. 1b, lane 14, Trx-NVL^167–216^). Thus, the MTR4-binding determinants of NVL reside in an unstructured region that is present in the human orthologue and more generally in chordate NVL proteins, but not in lower eukaryotes.

### NVL targets the MTR4 KOW domain

To identify where the MTR4-binding determinants for NVL reside, we performed GST pull down assays with MTR4 constructs harboring the DExH core of the helicase (GST-MTR4∆N∆arch) and the KOW domain (GST-MTR4 KOW). Neither Trx-NVL^1–266^ nor Trx-NVL^167–216^ co-precipitated with GST-MTR4-ΔNΔarch (Fig. 1b, lanes 12 and 15). In contrast, the KOW domain characteristic of the MTR4 arch (GST-MTR4-KOW) was able to co-precipitate both Trx-NVL^1–266^ and Trx-NVL^167–216^ (Fig. 1b, lanes 13 and 16). We quantified the strength of the interaction by biophysical approaches. Using microscale thermophoresis (MST), we measured a dissociation constant of ~1 µM between a YFP-tagged version of NVL^167–216^ and MTR4-KOW (Fig. 1c). A similar dissociation constant was obtained when testing the interaction of NVL^167–216^ and MTR4-∆N using isothermal titration calorimetry (ITC) (Supplementary Fig. 1d). The fact that the KOW domain alone binds NVL^167–216^ as strongly as MTR4-∆N indicated that the NVL^167–216^ binding region resides within the KOW domain.

**Fig. 2:**
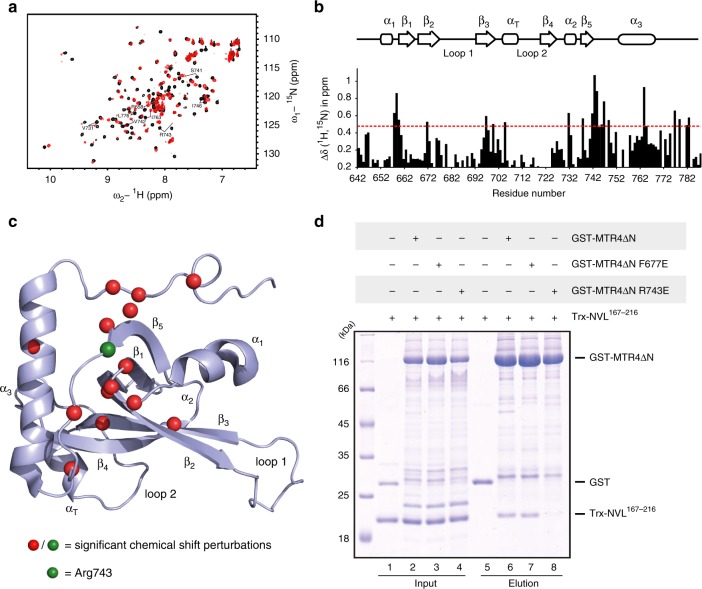
Analysis of MTR4 KOW-NVL complex by NMR and site directed mutagenesis. **a** Overlay of ^1^H-^15^N-HSQCs of either MTR4 KOW alone (black) or in complex with a six-fold excess of NVL2 (red). Selected residues experiencing large chemical shift perturbations (CSPs) are labeled. **b** Plot of CSPs per residue of the MTR4 KOW sequence. The red line marks the threshold of significant CSPs, which are mapped on the model of MTR4 KOW (PDB 6IEH) in panel c. Gaps indicate either prolines or residues that could not be assigned. **c** MTR4 KOW domain (PDB 6IEH) with labeled secondary structural elements as assigned by NMR and residues showing significant CSPs (displayed as red/green spheres). **d** Protein co-precipitations by pull down assays. GST-tagged MTR4∆N (WT or mutants) were incubated with Trx-NVL^167–216^ before co-precipitation with glutathione sepharose beads. A total of 3% of the input (lanes 1–4) and 30% of the eluates (lanes 5–8) were analyzed on 15% SDS-PAGE gels and visualized by staining with coomassie brilliant blue

We proceeded to characterize the NVL^167–216^-binding site of MTR4 with structural approaches. Using NMR spectroscopy, we confirmed that the MTR4-KOW domain has a sequential arrangement of secondary structure elements consistent with the fold observed in a recent crystal structure of an MTR4-NRDE-2 complex^31^, namely a five-stranded β-sheet flanked by a long C-terminal helix and containing smaller helical segments within loops (Supplementary Fig. 2). We then carried out titration experiments using a ^15^N-labelled MTR4-KOW protein and adding increasing amounts of unlabeled NVL^167–216^. In line with a dissociation constant of ~1 µM, we observed an intermediate exchange regime for most of the peaks during the titration in HSQC (heteronuclear single quantum coherence) spectra. Chemical shift perturbations (CSP) measured upon NVL2^167–216^ addition revealed significant effects (Fig. 2a–c) with clusters of strongly shifting peaks around residues 658, 695, 743, and 764. From the NMR analysis of the NVL^167–216^ - MTR4-KOW interaction, the CSPs were very similar to those we had previously reported for the yeast Nop53-Mtr4 KOW interaction^36^ (Supplementary Fig. 3a, 3b). Consistently, a reverse-charge substitution of Arg743 (R743E mutant) impaired the binding of both NVL^167–216^ (pull-down assays in Fig. 2d, lane 8) and of the human Nop53 orthologue (Supplementary Fig. 3c).

### Identification of W-AIM: a tryptophan arch-interacting motif of NVL

**Fig. 3:**
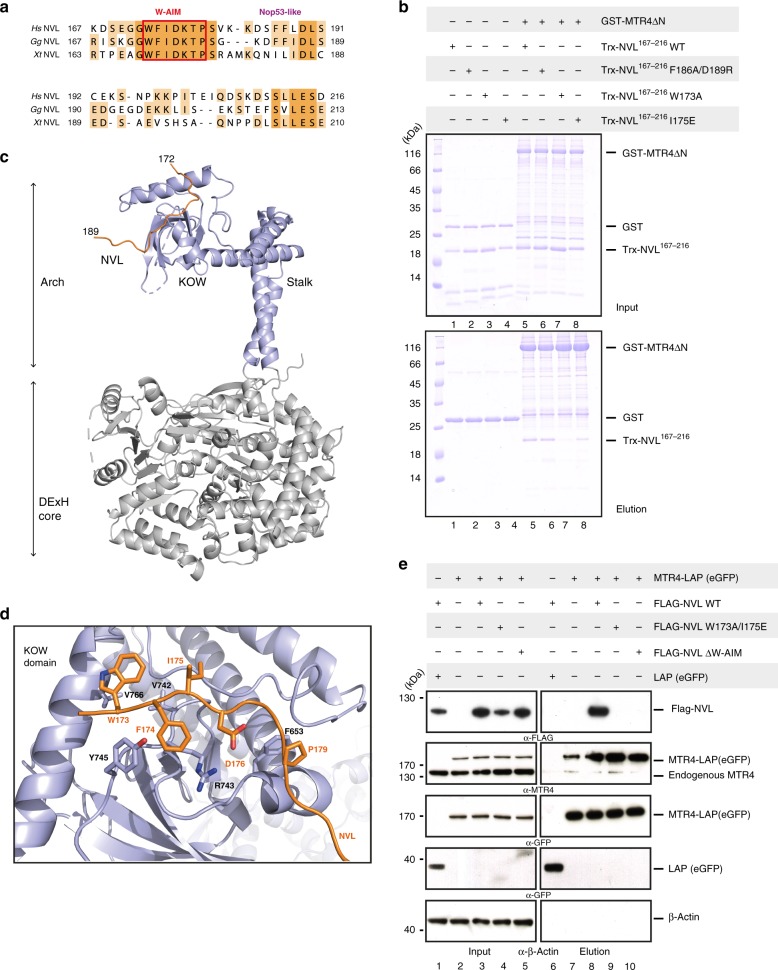
The vertebrate specific W-AIM in NVL is crucial for binding to MTR4 KOW. **a** Sequence alignment of vertebrate specific insertion regions of representative NVL sequences, *Homo sapiens* (*Hs*), *Gallus gallus* (*Gg*), *Xenopus tropicalis* (*Xt*), highlighting the W-AIM (GWFIDKTP) (red box), and the Nop53-like region (LFXϕD) (purple). The sequences were obtained from the UniProt database and aligned using the T-coffee server^59^. **b** Protein co-precipitations by pull down assays. GST-tagged MTR4∆N was incubated with either wild type Trx-NVL^167–216^ or its indicated mutant before co-precipitation with glutathione sepharose beads. A total of 3% of the input (top) and 30% of the eluates (bottom) were analyzed on 15% SDS-PAGE gels and visualized by staining with coomassie brilliant blue. **c** Overall structure of MTR4∆N_2_^70–1042^ - NVL^167–216^ complex, with the DExH core of MTR4 colored in gray and arch colored in light blue. NVL is colored in orange. **d** Zoom-in view of the interactions between MTR4 KOW (light blue) domain and NVL (orange). Domains are colored as in Fig. 3a and viewed are 90° rotation along the horizontal and vertical axes with respect to the view in Fig. 3a. Residues discussed in the text are highlighted and labeled. **e** Cellular co-IP assay. FLAG-tagged wild type NVL construct, or its indicated mutant variant, were transiently expressed in cells stably expressing MTR4-LAP. After precipitation of MTR4-LAP, a total of 0.5% of the input (left) and 8.0% of the eluates (right) were analyzed on 4–12% SDS-PAGE gel followed by western blotting analysis. The primary antibody used is indicated below the panel

Given that NVL^167–216^ binds the KOW domain of MTR4 similarly to Nop53, we expected the presence of a similar AIM motif. A stretch of amino acids within this segment (NVL residues 185–190) appeared to show significant sequence similarity to the Nop53 AIM sequence (Fig. 3a). Surprisingly in this context, the F186A and D189R mutations in the Nop53-like stretch did not affect NVL^167–216^ binding to MTR4 in a pull-down assay with recombinant proteins (Fig. 3b, compare lanes 5 and 6), indicating that this segment is not a bona-fide AIM. To identify the arch-interacting motif in NVL^167–216^, we analyzed the sequences from different species in terms of evolutionary conservation. We noticed that vertebrates feature a conserved hydrophobic segment GWFIDKTP (residues 172–179, Fig. 3a; Supplementary Fig. 1a). Mutations in this segment (W173A, I175E) either abolished or impaired NVL^167–216^ binding to MTR4∆N in pull-down assays in vitro (Fig. 3b, lanes 7 and 8), suggesting that this stretch functions as an arch-interacting motif, which we coin ‘tryptophan-centered arch - interacting motif’ (W-AIM).

We co-crystallized the NVL W-AIM peptide in complex with a construct of human MTR4 encompassing both the DExH core and the arch domain (residues 70–1042) (Table 1). The overall structure of the helicase is similar to that of the human NRDE-2-MTR4 complex^31^, but with a notably different conformation of the arch domain. In the NVL-bound crystal structure, the arch domain of MTR4 is an open conformation, with the KOW domain clearly separated from the helicase core (Fig. 3c and Supplementary Fig. 4a). The NVL W-AIM polypeptide chain binds in an extended conformation, similar to that reported for NRDE-2^31^, Nop53^36^ and Air2^34^ (Supplementary Fig. 4b, 4c). The hydrophobic side chains of Trp173_NVL_, Phe174_NVL_, and Ile175_NVL_ contact Val766_MTR4_, Tyr745_MTR4_, and Val742_MTR4_ respectively, while Asp176_NVL_ interacts with Arg743_MTR4_ (Fig. 3d). The NVL polypeptide chain then forms a pronounced bend at Pro179_NVL_ (which contacts Phe653_MTR4_) and continues to form a crystal contact, docking at the Trf4-binding site of a symmetry related helicase core (Supplementary Fig. 5a). Consistent with the structural data, mutation of Asp176_NVL_ (D176A mutant) impairs the binding of NVL^167–216^ to MTR4∆N in a GST pull down experiment (Supplementary Fig. 5b).

**Table 1:** Data collection and refinement statistics

	Mtr4ΔN^70–1042^ – NVL^167–216^
Data collection	
Space group	P6_1_
Cell dimensions	
* a*, *b*, *c* (Å)	184.37, 184.37, 90.53
α, β, γ (°)	90.0, 90.0, 120.0
Resolution (Å)	92.18–3.07
* R*_sym_ or *R*_merge_	15.7 (527.6)^a^
* I* / σ*I*	12.6 (0.9)
Completeness (%)	99.9 (99.5)
Redundancy	16.5
CC_1/2_	99.9 (62.7)
Refinement	
Resolution (Å)	60.35–3.07 (3.22–3.07)
No. reflections	32,919
* R*_work_/*R*_free_	22.3 / 25.7
No. atoms	
Protein	7074
Ligand/ion	58
Water	6
* B*-factors	
Protein	123.57
Ligand/ion	138.03
Water	92.34
R.m.s. deviations	
Bond lengths (Å)	0.003
Bond angles (°)	0.478

^a^values in parentheses are for highest-resolution shell

Finally, we tested the effect of the MTR4-interacting residues of NVL in human cells. To this end, we carried out co-immunoprecipitation (co-IP) assays in HeLA cells stably expressing MTR4 with an eGFP ‘localization and affinity purification’ (LAP) tag at the N-terminus and transiently expressing FLAG-tagged full-length NVL constructs (wild type, or with the W173A/I175A mutation or with deletion of the entire hydrophobic segment). Western blotting analysis confirmed that wild type NVL bound MTR4 and that disruption of the 172–180 segment, either by mutation or deletion, impaired the NVL-MTR4 interaction (Fig. 3e, compare lanes 8, 9, 10). Taken together, we conclude that NVL interacts with the KOW domain of MTR4 using a short linear W-AIM sequence. The NVL W-AIM is more hydrophobic than the Nop53 AIM^35^, rationalizing why it binds MTR4 with an order of magnitude higher affinity than the yeast Nop53-Mtr4^36^ and human Nop53-MTR4 interactions (Supplementary Fig. 3d).

### The unstructured region of ZCCHC8 interacts with the MTR4 KOW domain

**Fig. 4:**
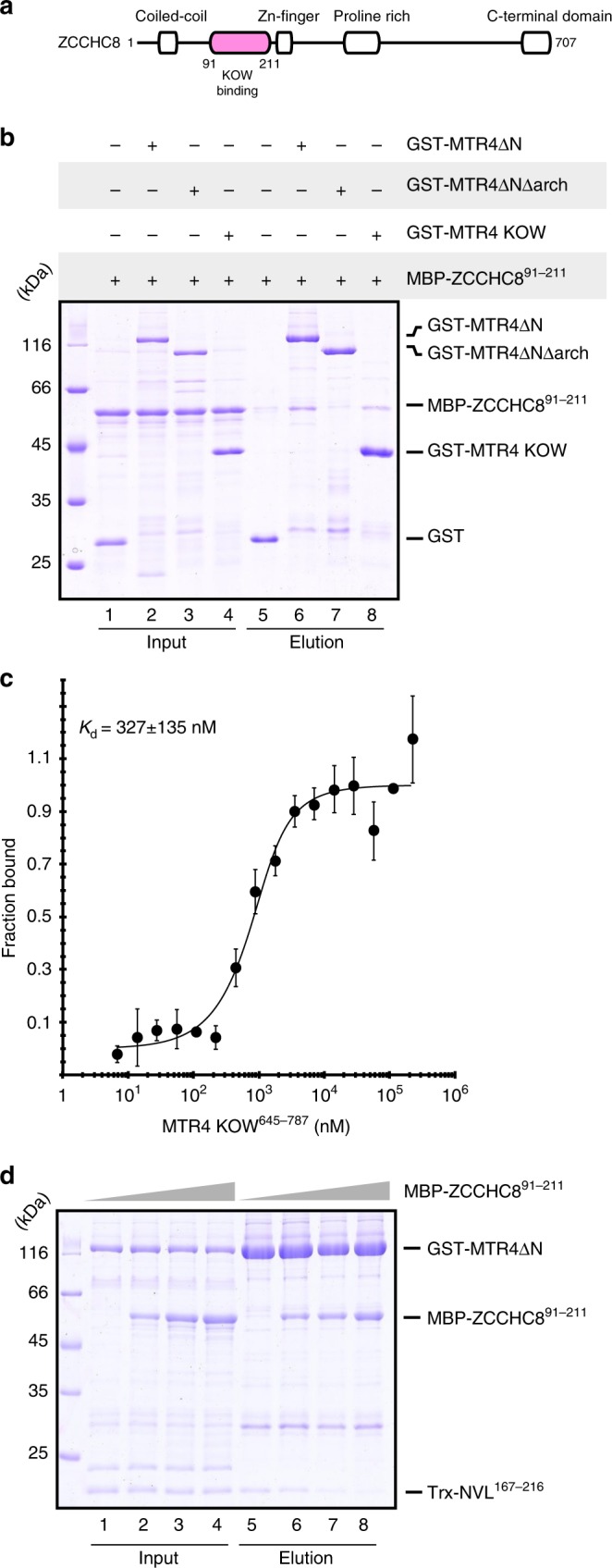
The N-terminus of ZCCHC8 interacts with the MTR4 KOW domain. **a** Schematic representation of the domain organization of ZCCHC8. Domain boundaries are obtained from previous studies^37,43^ and computational sequence analysis. **b** Protein co-precipitations by pull down assays. GST-tagged MTR4∆N, MTR4∆N∆arch, and MTR4 KOW were incubated with MBP-ZCCHC8 ^91–211^ before co-precipitation with glutathione sepharose beads. A total of 3% of the input (lanes 1–4) and 30% of the eluates (lanes 5–8) were analyzed on 12% SDS-PAGE gels and visualized by staining with coomassie brilliant blue. **c** Microscale thermophoresis experiment with MTR4 KOW and MBP-ZCCHC8 ^91–211^-(GS)_3_-eYFP. In all, 1 μM MBP-ZCCHC8 ^91–211^-(GS)_3_-eYFP was incubated with increasing concentrations of MTR4 KOW and thermophoresis was measured by tracking the fluorescence of the ZCCHC8-YFP fusion protein. The binding curve was calculated using MO software (Nanotemper technologies) and the dissociation constant (*K*_d_) is given in the inset. The error bars represent the standard deviations of each data point calculated from three independent thermophoresis measurements. **d** Competition experiment using GST pull down assay technology. A preformed GST-MTR4∆N:Trx-NVL^167–216^ complex was incubated with increasing concentrations of MBP-ZCCHC8^(91–211)^ before precipitation with glutathione sepharose beads. A total of 3% of the input (lanes, 1–4) and 30% of the eluates (5–8) were analyzed on 15% SDS-PAGE gels and visualized by staining with coomassie brilliant blue

Identification of the W-AIM sequence in NVL motivated us to examine whether other known MTR4 interactors also contain a similar tryptophan-centered motif. One such interactor is ZCCHC8, the scaffolding subunit of the NEXT complex^27,37,43^. This modular protein contains a predicted N-terminal coiled–coil domain (residues 40–80), a Zinc-knuckle domain (residues 222–246), a proline-rich domain (residues 287–334) that interacts with RBM7^43^ and a C-terminal domain (CTD) (residues 659–707) (Fig. 4a) that interacts with the DExH core of MTR4 and activates it^37^. The N-terminal portion of ZCCHC8 is expected to contain an additional MTR4-binding site^37^. Within the N-terminal portion, we focused our attention on the linker between the coiled-coil and the Zinc-knuckle domains, as it appeared to contain an NVL-like tryptophan-containing sequence (Supplementary Fig. 6a). We expressed a large portion of this linker (residues 91–211) tagged to maltose binding protein (MBP-ZCCHC8^91–211^) and purified the resulting construct for pull-down assays with the versions of GST-tagged MTR4 described above (Fig. 1a). MBP-ZCCHC8^91–211^ co-precipitated with GST-MTR4-∆N but not with GST-MTR4-∆N∆arch (Fig. 4b, lanes 6 and 7). Similar to the results we had obtained for NVL^167–216^, the pull-down assays showed that MBP-ZCCHC8^91–211^ interacts with GST-MTR4-KOW (Fig. 4b, lane 8). Next, we determined the affinity of the MTR4-KOW-ZCCHC8^91–211^ interaction using microscale thermophoresis (MST) with a fluorescent-tagged version of ZCCHC8^91–211^ (that we had engineered by fusing a YFP to the C-terminus). In this quantitative assay, we measured a dissociation constant (*K*_D_) of ~0.3 µM (Fig. 4c), indicating a higher affinity than that of the NVL^167–216^ fragment. To corroborate these results, we pre-formed a GST-MTR4-∆N - Trx-NVL^167–216^ complex and incubated it with increasing amounts of MBP-ZCCHC8^91–211^ before subjecting the mixtures to GST pull-down assays. The competition assay showed that ZCCHC8^91–211^ could displace NVL^167–216^ from the pre-formed complex (Fig. 4d, lanes 5–8), suggesting that they interact with the exosome helicase in a mutually exclusive manner.

### Identification of C-AIM: a cysteine arch-interacting motif of ZCCHC8

**Fig. 5:**
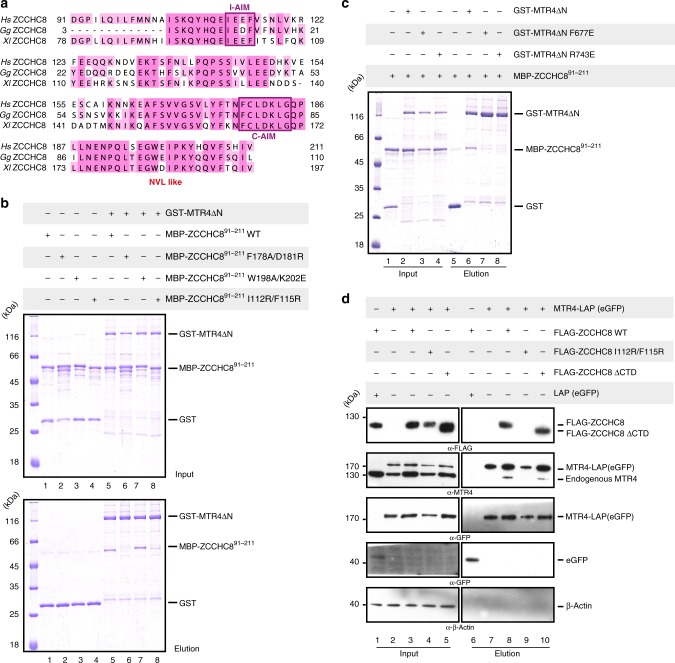
Analysis of the ZCCHC8-MTR4 KOW complex by site directed mutagenesis. **a** Sequence alignment of the region between the predicted coiled coil domain and the zinc finger of ZCCHC8 from representative metazoan species, *Homo sapiens* (*Hs*), *Gallus gallus* (*Gg*), *Xenopus laevis* (*Xl*), highlighting NVL-like region (red), Nop53-like AIM (C-AIM) and ZCCHC8 specific I-AIM (purple boxes). The sequences were obtained from the UniProt database and aligned using the T-coffee server^59^. **b** Protein co-precipitations by pull down assays testing ZCCHC8 ^91–211^ mutants for MTR4∆N binding ability. GST-tagged MTR4∆N was incubated with either ZCCHC8^91–211^ WT or mutants before co-precipitation with glutathione sepharose beads. A total of 3% of the input (top) and 30% of the eluates (bottom) were analyzed on 12% SDS-PAGE gels and visualized by staining with coomassie brilliant blue. **c** Protein co-precipitations by pull down assays testing MTR4∆N mutants for their ZCCHC8 ^91–211^ binding ability. GST-tagged MTR4∆N (WT or mutant variants) were incubated with MBP-ZCCHC8 ^91–211^ before co-precipitation with glutathione sepharose beads. A total of 3% of the input (lanes, 1–4) and 30% of the eluates (5–8) were analyzed on 12% SDS-PAGE gels and visualized by staining with coomassie brilliant blue. **d** Cellular co-IP assay. FLAG-tagged ZCCHC8 constructs (WT/IF mutant/CTD deletion) were transiently expressed in cells stably expressing MTR4-LAP. After precipitation of MTR4 taking advantage of the LAP tag, a total of 0.5% of the input (left) and 8.0% of the eluates (right) were analyzed on 4–12% SDS-PAGE gel followed by western blotting analysis. The primary antibody used is indicated below the panel

We proceeded to identify the MTR4-binding site within the N-terminal region of ZCCHC8. We first interrogated the NVL-like tryptophan-containing patch (GWEIPK, residues 197–202, Fig. 5a). Surprisingly, mutation of Trp198 and Lys202 (W198A/K202E mutant) did not alter the interaction of ZCCHC8^91–211^ with MTR4-∆N in GST pull down assays (Fig. 5b, lane 7), indicating that this patch of ZCCHC8 is not a bona-fide W-AIM sequence.

To identify the arch-interacting motif in ZCCHC8^91–211^, we took a similar bioinformatics approach as described above for NVL. When analyzing the evolutionary conservation of ZCCHC8^91–211^, we identified a conserved patch centered at the FCLDKLG segment (residues 178–184, Fig. 5a). Mutation of Phe178 and Asp181 (F178A/D181R mutant) in this segment impaired the interaction with MTR4-∆N (Fig. 5b, lane 6). This cysteine-centered arch-interacting motif (C-AIM) is loosely related to the Nop53 AIM and the NVL W-AIM segments, and is thus expected to bind to the same site of the KOW domain. Consistently, the Arg743 reverse-charge substitution (R743E) in MTR4 also impaired the binding of ZCCHC8^91–211^ (Fig. 5c, lane 8).

### ZCCHC8 also contains a non-canonical arch-interacting motif: I-AIM

While attempting to narrow down the KOW binding region of ZCCHC8 further, we observed that N-terminal truncation of ZCCHC8^91–211^ resulted in a near loss of MTR4 binding (Supplementary Fig. 6b). Based on these results, we reasoned that ZCCHC8^91–211^ might harbor an additional MTR4-binding motif that would be predicted to bind to an adjacent surface on the KOW domain (and thus show no resemblance to the previously identified arch-interacting motifs). Using bioinformatics approaches, we identified another conserved segment upstream of C-AIM (IEEF, residues 112–115). Indeed, mutation of Ile112 and Phe115 (I112R/F115R mutant) severely weakened the MTR4-∆N - ZCCHC8^91–211^ interaction in GST pull-down assays (Fig. 5b, lane 8). Thus, this segment (which we refer to as an isoleucine-centered arch-interacting motif, or I-AIM) also contributes to MTR4 binding. Next, we mapped the possible MTR4-binding site of I-AIM. The β-barrel of the MTR4 KOW domain is structurally related to that of Tudor domains (Supplementary Fig. 7a), small globular folds that generally present a hydrophobic pocket for binding methylated arginines and lysines^44^. Although there is little overall sequence similarity, MTR4-KOW features hydrophobic residues at the equivalent position of the substrate–binding residues of Tudor domains (Supplementary Fig. 7b)^44,45^. In particular, MTR4 Phe677 is evolutionarily conserved (Supplementary Fig. 7b) and is located on a surface adjacent to the Arg743 site where the Nop53 like AIM, W-AIM and C-AIM sequences are recognized (Supplementary Fig. 7c). In line with this site being used for the additional I-AIM sequence of ZCCHC8^91–211^, mutation of Phe677 (F677E) disrupted binding of ZCCHC8^91–211^ (Fig. 5c, lane 7) and did not affect the binding of NVL^167–216^ (Fig. 2d, lane 7) or human Nop53 (Supplementary Fig. 3c, lane 7).

We tested the importance of the I-AIM segment in human cells. HeLa LAP-MTR4 cells were transfected with FLAG-tagged full-length ZCCHC8 constructs (wild type, the I-AIM I112R/F115R double mutant or a deletion construct lacking the ZCCHC8 CTD). Western blotting analysis of the resulting co-IPs revealed that disrupting the I-motif by mutation is sufficient to impair the ZCCHC8-MTR4 interaction, whereas deletion of the CTD did not significantly affect complex formation (Fig. 5d, lanes 8, 9, 10). These mutations also did not affect the ZCCHC8-RBM7 interaction (Supplementary Fig. 6c). The CTD of ZCCHC8 has been shown to bind the DExH core of MTR4 and to enhance helicase activity^37^. In contrast, we found that neither the ZCCHC8 nor the NVL AIM motifs have a significant effect on the catalytic properties of MTR4, as judged by assaying both ATP hydrolysis and RNA helicase activities (Supplementary Fig. 8). These results suggest a division of labor of the N-terminal and C-terminal ZCCHC8 regions: while the C-terminal domain regulates the activity of the helicase^37^, the N-terminal region plays has a central scaffolding role in incorporating MTR4 into the NEXT complex.

## Discussion

In this study, we show that the human nuclear exosome adaptors NVL and ZCCHC8 bind the MTR4 KOW domain on a surface that is also employed by Nop53^36^ and NRDE-2^31^ using distinct arch-interacting motifs^35^. The AIMs of Nop53/Utp18/Air2 (LFxϕD(x)_1–2_G/P), NVL (GWFIDKTP), ZCCHC8 (NFCLDKLG), and NRDE-2 (SFRTDKKP) can best be considered as subfamilies of canonical AIMs. With insight, sequences in both NVL and ZCCHC8 that at first glance appeared to resemble known AIMs instead contain individual amino acids that are likely to prevent MTR4 binding. The consensus sequence of canonical bona-fide AIMs can thus be re-defined as xωxxD(x)_1/2_G/P, with a C-terminal glycine or proline residue that allows the polypeptide chain to bend as it extends away from its binding site. The aromatic (ω) and polar/non-polar amino acids might vary, but tend to be conserved within individual subfamilies of canonical AIMs. Despite the variability, all canonical AIM sequences are recognized, in a mutually exclusive manner, at a surface pocket of the MTR4 KOW domain that is defined by the presence of Arg743 (yeast Mtr4 Arg774). Mutation of this surface pocket may thus be a useful tool to probe new MTR4-interacting proteins that contain arch-interacting motifs, as these motifs are difficult to identify due to the degeneracy of their consensus sequence. Furthermore, we found that ZCCHC8 harbors an additional arch-interacting motif that does not conform to the canonical AIM consensus sequences. These findings show how different exosome adaptor proteins have evolved similar mechanisms to recognize MTR4 in a specific and mutually exclusive manner, but can also modulate the affinity and thus selectivity with which they are ultimately recruited to the nuclear exosome.

## Methods

### Protein expression and purification

Human MTR4 constructs (full-length, MTR4-∆N (75–1042), MTR4-KOW (645–787) and MTR4-∆N∆arch, where residues 598–842 were substituted by 2xGS linker) were expressed as 6xHis-GST-tagged fusion (cleavable with 3 C protease) proteins in BL21 star (DE3) *E*. *coli* cells, grown either in terrific broth (for biochemical studies) or minimal medium supplemented with ^15^N labelled ammonium chloride and/or ^13^C labeled Glucose (for NMR studies). The proteins were purified using a Ni-nitrilotriacetate (NTA) affinity column and a heparin column (GE healthcare) for ion exchange chromatography. When appropriate, the His-GST tag was cleaved at this point by incubation with 3 C protease, followed by removal of the tag with an additional Ni-NTA affinity step. Finally, the protein was subjected to size exclusion chromatography on a Superdex 200 column (GE healthcare) in 50 mM Hepes/NaOH pH 7.5, 150 mM NaCl, 5% (v/v) glycerol, 2 mM DTT.

The human MTR4 construct used for crystallization (71–1042) was cloned as GST fusion construct with a TEV cleavage site immediately preceding the MTR4 sequence which when cleaved would leave a glycine (the natural 70th residue in MTR4) yielding MTR4 protein with residues 70–1042 (referred to as MTR4∆N_2_ in the text). The construct was purified as described above.

The primers used for cloning all the constructs described are listed in Supplementary table 1.

All NVL and ZCCHC8 constructs and truncation mutants described in the text were expressed as 6xHis-Trx and 6xHis-MBP tagged fusion (cleavable with 3 C protease) proteins, respectively in BL21 star (DE3) E. *coli* cells. The proteins were purified using a Ni-NTA affinity column. When appropriate, the tags were cleaved upon incubation with 3 C protease at this step, followed by an additional Ni-NTA affinity step for the removal of the tag. In case of ZCCHC8, the tag cleavage was performed only in the presence of MTR4 to prevent the protein from precipitating. In the final purification step, the proteins were subjected to size-exclusion chromatography on a Superdex 75 in 50 mM Hepes/NaOH pH 7.5, 150 mM NaCl, 2 mM DTT.

### Biophysical assays

The microscale thermophoresis measurements were performed on a NanoTemper Monolith NT.115 machine. Before the measurements, all samples were dialyzed against a buffer containing in 50 mM Hepes/NaOH pH 7.5, 150 mM NaCl, 5% (v/v) glycerol, 0.5 mM TCEP. For NVL, 50 nM of Trx-NVL^167–217^-(GS)_3_-eYFP was incubated with increasing concentrations of unlabeled MTR4 KOW and thermophoresis was measured with an MST power of 20% and an LED power of 20%. For ZCCHC8, 1 μM of MBP-ZCCHC8^91–211^ -(GS)_3_-eYFP was incubated with increasing concentrations of unlabeled MTR4-KOW and thermophoresis was measured with an MST power of 70% and an LED power of 15%. In both cases, the MTR4 KOW concentration series was produced by serial dilution (1:1). Titrations were performed in triplicates and the data were analyzed using the Thermophoresis with T-Jump strategy option with the MO software (NanoTemper Technologies).

Isothermal calorimetry experiments were carried out using the ITC200 Isothermal titration calorimeter from Microcal. Before the measurement, all samples were dialysed against a buffer containing 50 mM Hepes/NaOH pH 7.5, 150 mM NaCl, and 0.5 mM TCEP. For NVL: MTR4-∆N (the reactant) samples were concentrated to 40 µM and NVL (the injectant) to 500 µM. For human NOP53: MTR4-KOW (the reactant) samples were concentrated to 100 µM and NOP53 (the injectant) to 1 mM. Titrations were carried out at 25 °C with 2 µL of the injectant per injection added to 200 µL of reactant cell solution. All data were processed and curves fitted using Origin5.0.

### Biochemical assays

For pull-down assays, appropriate protein mixtures were incubated in 50 mM Hepes (pH 7.5), 150 mM NaCl, 5% (v/v) glycerol, 0.05% (v/v) NP40, 1 mM DTT for 30 min at 4 °C. For ZCCHC8, 2 μM GST-MTR4∆N was incubated with 8 μM MBP-ZCCHC8 constructs. For NVL, 10 μM GST-MTR4∆N was incubated with 20 μM Trx-NVL constructs. For NOP53, 30 μM GST-MTR4∆N was incubated with 60 μM Trx-NOP53^84–123^ in a total volume of 30 μl. The protein mixtures were then incubated with Glutathione sepharose beads (GE healthcare) for 2 h. Post incubation, the beads were washed three times with 0.1 ml incubation buffer and the retained material was eluted with incubation buffer supplemented with 30 mM reduced glutathione. Input material (1–3%) and eluates (~30%) were analyzed by SDS-PAGE and Coomassie staining.

For ATPase assays, 150 pmol of MTR4∆N or MTR4∆N-containing complexes were incubated 40 nmol MESG (2-amino-6-mercapto-7-methylpurine ribonucleoside) and 0.5 U purine nucleoside phosphorylase (Enzchek Phosphate Assay kit, Invitrogen) in a buffer containing 50 mM MOPS pH 6.5, 50 mM Nacl, 2.5 mM MgCl_2_, 5 mM β-mercaptoethanol and 5% (v/v) glycerol. For reactions containing RNA, the mixture was incubated with 2 μg poly-U RNA (Sigma). The reaction was initiated by addition of ATP to a final concentration of 1 mM. The generation of 2-amino-6-mercapto-7-methylpurine from MESG was monitored by measuring absorbance increase at 360 nm on a plate reader (Infinite M1000 Pro, Tecan) for 12 min at 60 s intervals. The data were normalized by subtracting the y-intercept from the raw data. The experiment was performed in triplicate. The mean (*n* = 3) and standard deviation (error bars) were plotted using Graphpad prism 8.

Helicase assays were performed essentially as described by Puno et al.^37^. A duplex RNA was formed by mixing a 5’ Fluorescein amidite (FAM) labeled RNA (FAM-AGCACCGUAAAGACGC) with 1.5 molar excess of complementary RNA with a 25 A overhang (GCGUCUUUACGGUGCUAAAAAAAAAAAAAAAAAAAAAAAAA) in a buffer containing 20 mM Tris-HCl pH 7.5 and 50 mM NaCl. To anneal the RNA duplex, the mixture was heated to 95 °C and allowed to cool down slowly to ambient temperature by turning off the heat block. 0.3 pmol of duplex RNA was incubated with 3.75, 7.5, 30 pmol of MTR4∆N or MTR4∆N- containing complexes in a buffer containing 50 mM MOPS pH 6.5, 50 mM Nacl, 0.5 mM MgCl_2_, 5 mM β-mercaptoethanol and 5% (v/v) glycerol for 5 min at 30 °C. The reaction was then initiated by the addition of ATP, MgCl_2_ and a trap DNA oligo complementary to the FAM labeled RNA (GCGTCTTTACGGTGCT) to a final concentration of 2 mM, 2 mM and 400 nM respectively. The reactions were quenched after 40 min by placing the tubes on ice and adding quenching buffer to a final concentration of 0.5% (w/v) SDS, 10 mM EDTA, 10% (v/v) glycerol and 0.005% (w/v) xylene cyanol. The sample were analyzed by electrophoresis on a 15% acrylamide–Tris base, boric acid, EDTA (TBE) gel. The fluorescence was imaged using a Typhoon FLA 7000. The oligonucleotides used in the assay were obtained from Ella Biotech GmBH.

### NMR spectroscopy

NMR measurements of MTR4-KOW were performed in phosphate buffered saline (10 mM PO_4_^3−^, 137 mM NaCl, 2.7 mM KCl) mixed with 10% (v/v) D_2_O. Backbone chemical shift assignments of the KOW region were obtained from two ^13^C, ^15^N-labelled samples with protein concentration of 700 μM and 500 μM, respectively. HNCA, HNCACB, HNcoCA, HNCO, HNcaCO, and 3D ^15^N-edited NOESY spectra^46^ were acquired at 298 K on Bruker Avance III spectrometers at field strengths corresponding to 600 and 800 proton Larmor frequency, equipped with TCI cryogenic probe heads. The ^15^N steady-state heteronuclear {^1^H}-^15^N NOE experiment was performed at 170 μM and a field strength of 500 MHz as described previously^47^. Protein binding was measured from HSQC experiments containing water-flip-back/WATERGATE^48,49^ sequences. Titrations with the NVL peptide were carried out at 298 K with a KOW concentration of 53 μM and in presence of 0.25, 0.5, 1, 2, and 6 stoichiometric molar equivalents of NVL. Spectra were recorded and processed with Topspin3.5 and analyzed with CCPNMR Analysis 2.4^50^ and Sparky (http://www.cgl.ucsf.edu/home/sparky). The chemical shift perturbations were calculated as CSP (ppm) = [6(ΔH)^2^ + (ΔN)^2^]^0.5^.

### Crystallization and structure determination

MTR4∆N_2_ (70–1042) was mixed with 2 molar excess of NVL (167–216) in a buffer containing 20 mM Tris pH 7.5, 150 mM Nacl, 1 mM Mgcl_2_, 2 mM ADP and 1 mM TCEP. The crystallization trials were performed using a vapour diffusion setup. Initial crystals were obtained in the A4 (2 M ammonium sulfate, 0.1 M Tris pH 8.5) condition of SG1^TM^ screen (Molecular dimensions). The best diffracting crystals were obtained at a concentration of 10 mg/ml at 277 K in 0.1 M Tris pH 8, 1.8 M ammonium sulfate. The crystals were cryo-protected with reservoir solution supplemented with 30% glycerol prior flash-freezing in liquid nitrogen. Data were collected at 100 K at PXIII beamline of the Swiss light Source (Villigen, Switzerland). Data processing and scaling was performed using Xia2/DIALS^51,52^ within CCP4i2 software suite^53^. The crystals belong to the hexagonal spacegroup 169 (P6_1_) containing one molecule (MTR4∆N_2_ – NVL^167–216^) in the asymmetric unit and diffract to 3.07 Å resolution. The structure was solved by molecular replacement with Phaser^54^ within Phenix using the co-ordinates of the DExH core (98-593 and 847–1042) and KOW domain (645–787) of MTR4 (PDB 6IEH)^31^ as search models. The model was manually completed in COOT^55^ and refined using phenix.refine^56^. The optimal TLS groups for TLS refinement were determined using TLSMD server^57^. 96.1% of the protein backbone dihedral angles in the final model are in Ramachandran favored region. The figures of crystallographic models were prepared using pyMOL (Schrödinger, LLC).

### Cell culture and co-immunoprecipitation assays

Human HeLa Kyoto LAP, MTR4-LAP, RBM7-LAP cell lines were generated as outlined by Poser et al.^58^ and used for example by Lubas et al.^27^ (RBM7-LAP) and Meola et al.^30^ (MTR4-LAP). Briefly, HeLa Kyoto LAP MTR4-LAP, RBM7-LAP cells were cultured in DMEM + 10% FBS. Transfection was carried out using 10 µg of plasmid containing FLAG-tagged NVL or ZCCHC8 constructs, with Lipofectamine 2000 (Thermo Fisher) following the manufacturer’s instructions. Forty-eight hours after transfection the cells were collected and resuspended in extraction buffer (150 mM NaCl, 20 mM HEPES pH 7.4, 0.5% (v/v) Triton X-100) containing Protease Inhibitors (Roche). The lysates were sonicated twice for 5 s at 20 W, and cell debris was removed by centrifugation at 10,000 g for 10 min. Lysates were incubated 1 h with Dynabeads M-270 Epoxy (Invitrogen) coupled to a polyclonal llama anti-GFP antibody. Beads were washed three times in extraction buffer, then incubated 20 min at 25 °C with 100 units of Benzonase (Sigma) and 2 mM MgCl_2_. Beads were then washed twice in extraction buffer and proteins were eluted using NuPage LDS Sample Buffer (Invitrogen) at 70 °C for 10 min, and NuPage Sample Reducing Agent was added. The input material (0.5%) and the eluate (8.0%) were analyzed by SDS-PAGE on a NuPage Novex 4–12% Bis-Tris gel (Invitrogen). Western blotting analysis was performed following standard protocols. Following primary antibodies were used: anti-FLAG M2 (Dilution 1:50000; Sigma, F1804), anti-MTR4 (Dilution 1:4000; Abcam, ab70551), anti-GFP (Dilution 1:1000; Santa Cruz Biotechnology, SC-9996), anti-β-actin (Dilution 1:100000; Sigma, A2228) and anti-RBM7 (Dilution 1:1000; human protein atlas, HPA013993). Anti-mouse and anti-rabbit secondary antibodies coupled to Horseradish Peroxidase (Dako) were used.

### Reporting summary

Further information on research design is available in the Nature Research Reporting Summary linked to this article.

## Supplementary information

Supplementary Information

Peer Review

Reporting Summary

## Data Availability

A reporting summary for this Article is available as a Supplementary Information file. NMR backbone chemical shifts of the human MTR4 KOW domain were deposited at the BMRB under accession number 27831. The coordinates and the structure factors have been deposited in the Protein Data Bank with accession code PDB ID 6RO1. The source data are provided in Supplementary Fig. 10. All data is available from the corresponding author upon reasonable request.
