# Supplementary material for: The MTR4 helicase recruits nuclear adaptors of the human RNA exosome using distinct arch-interacting motifs
Source: Nat Commun. 2019 Jul 29;10:3393. doi: 10.1038/s41467-019-11339-x (PMC6662825; doi:10.1038/s41467-019-11339-x)
Supplement: Supplementary file 1 — Supplementary Information [file 41467_2019_11339_MOESM1_ESM.pdf]

SUPPLEMENTARY INFORMATION

The MTR4 helicase recruits nuclear adaptors of the human RNA exosome using distinct arch-interacting motifs

Lingaraju et al.

Supplementary Figure 1: Vertebrate specific N-terminal insertion in NVL interacts with MTR4

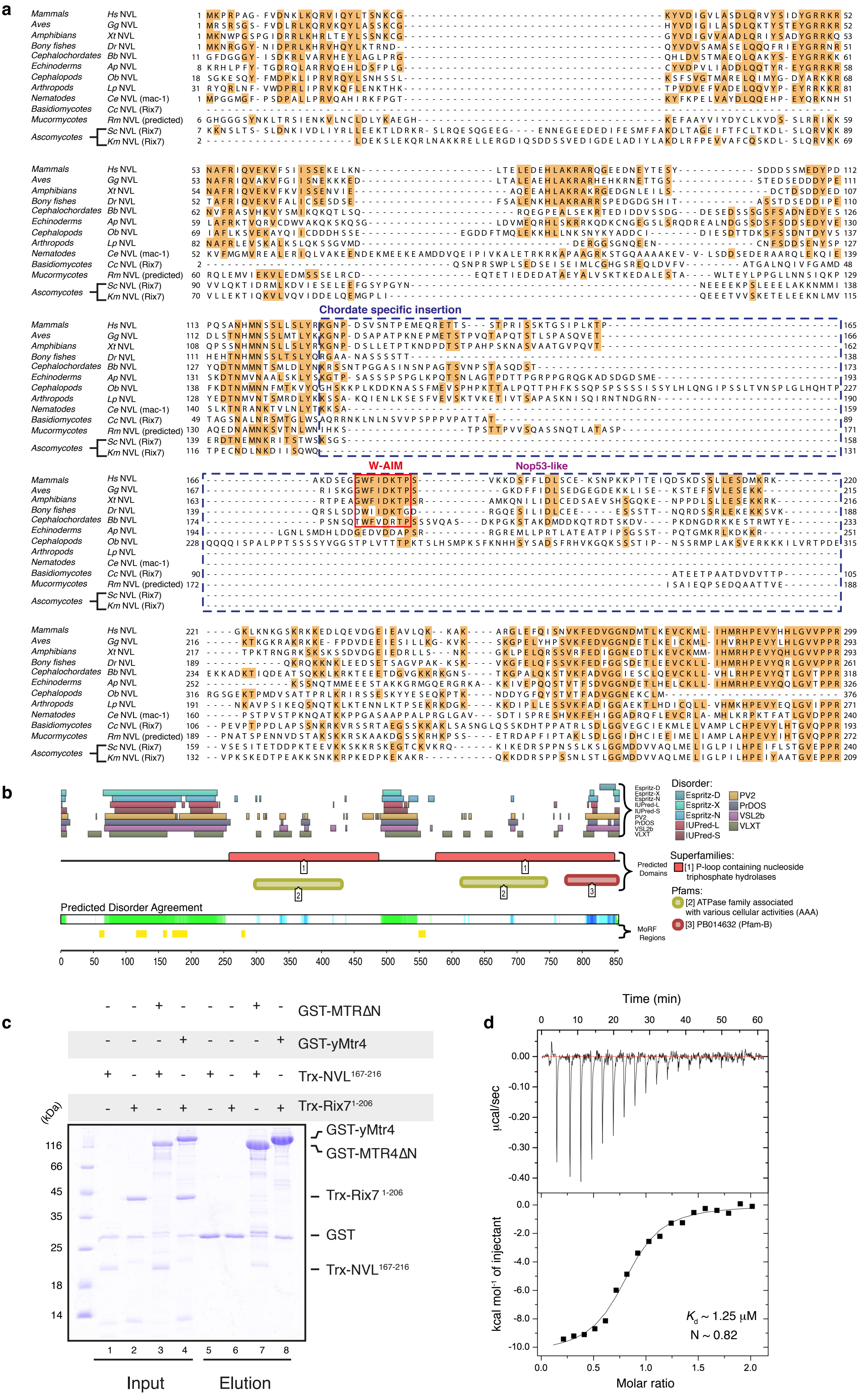

Supplementary Figure 1 (a) Sequence alignment of N-termini of representative vertebrate and fungal NVL sequences, highlighting the chordate specific insertion (blue dashed box), the W-AIM (red box) and a Nop53 (LFXϕD) like region (purple). The sequences were obtained from the NCBI database and aligned using the T-coffee server[1]. *Hs* stands for *Homo sapiens*, *Gg* for *Gallus gallus*, *Xt* for *Xenopus tropicalis*, *Dr* for *Danio rerio*, *Bb* for *Branchiostoma belcheri*, *Ap* for *Acanthaster planci*, *Ob* for *Octopus bimaculoides*, *Lp* for *Limulus polyphemus*, *Ce* for *Caenorhabditis elegans*, *Cc* for *Coprinopsis cineria*, Rm for *Rhizopus microsporus*, *Sc* for *Saccharomyces cerevisiae*, and *Km* for *Kluyveromyces marxianus*. (b) Disorder prediction of human NVL as obtained from the D^2P^2 database[2]. The region of NVL that is commonly predicted to be disordered by multiple algorithms is colored in bright green. (c) Protein co-precipitations by pull down assays. GST tagged MTR4ΔN or yMtr4 were incubated with the vertebrate specific NVL insertion, Trx-$NVL^{167\text{-}216}$ and the N-terminus of Rix7, Trx-$Rix7^{1\text{-}206,}$ respectively before co-precipitation with glutathione sepharose beads. A total of 3% of the input (left) and 30% of the eluates (right) were analyzed on 15% SDS-PAGE gels and visualized by staining with coomassie brilliant blue. (d) ITC experiment of MTR4ΔN with $NVL^{167\text{-}216}$. The filled squares show reference corrected titration of $NVL^{167\text{-}216}$ into the MTR4ΔN containing cell. The number of calculated binding sites (N), and dissociation constants (K_d) are shown in the inset.

Supplementary Figure 2: NMR analysis of the MTR4 KOW domain

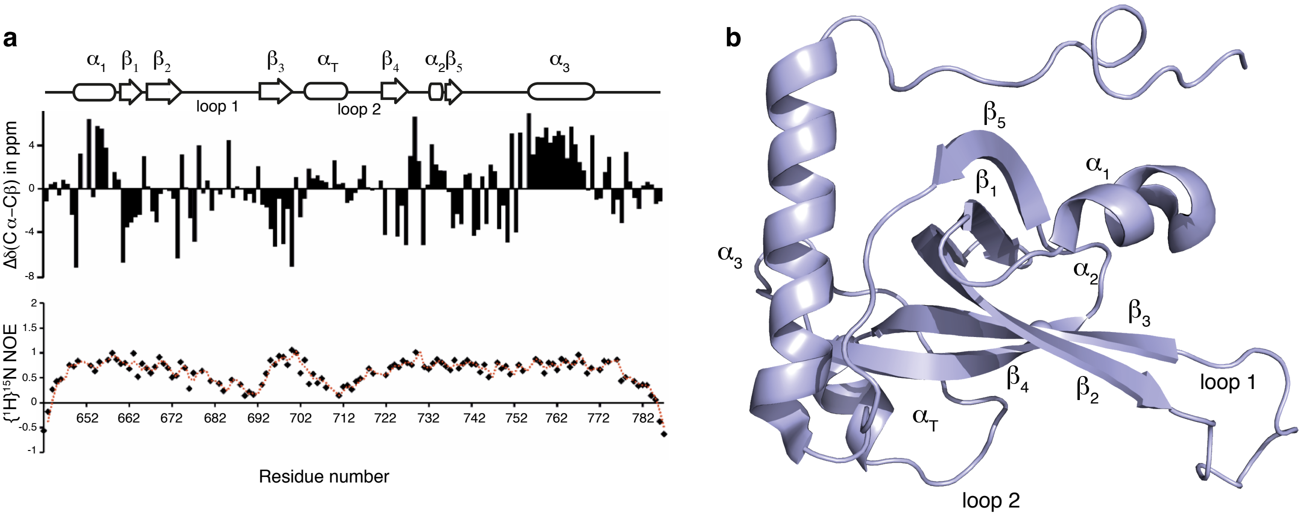

Supplementary Figure 2 (a) Upper bar chart shows secondary carbon chemical shifts of MTR4 KOW plotted against its primary sequence. The clusters of positive bars >2 represent α-helices and the clusters of negative bars >-2 represent β-strands. The scheme above shows a summary of secondary structure elements as derived from the analysis, and elements are labeled for comparison with panel b. The bottom chart shows a heteronuclear NOE plot of the MTR4 KOW demonstrating the residue-resolved rigidity along the primary sequence. Note that the two prominent dips are located within loops 1 and 2. (b) Secondary structure features of the KOW domain obtained from NMR analysis mapped on to the KOW domain from the crystal structure of MTR4 (PDB 6IEH)[3].

Supplementary Figure 3: NVL and Nop53 interact with MTR4 KOW in a similar manner

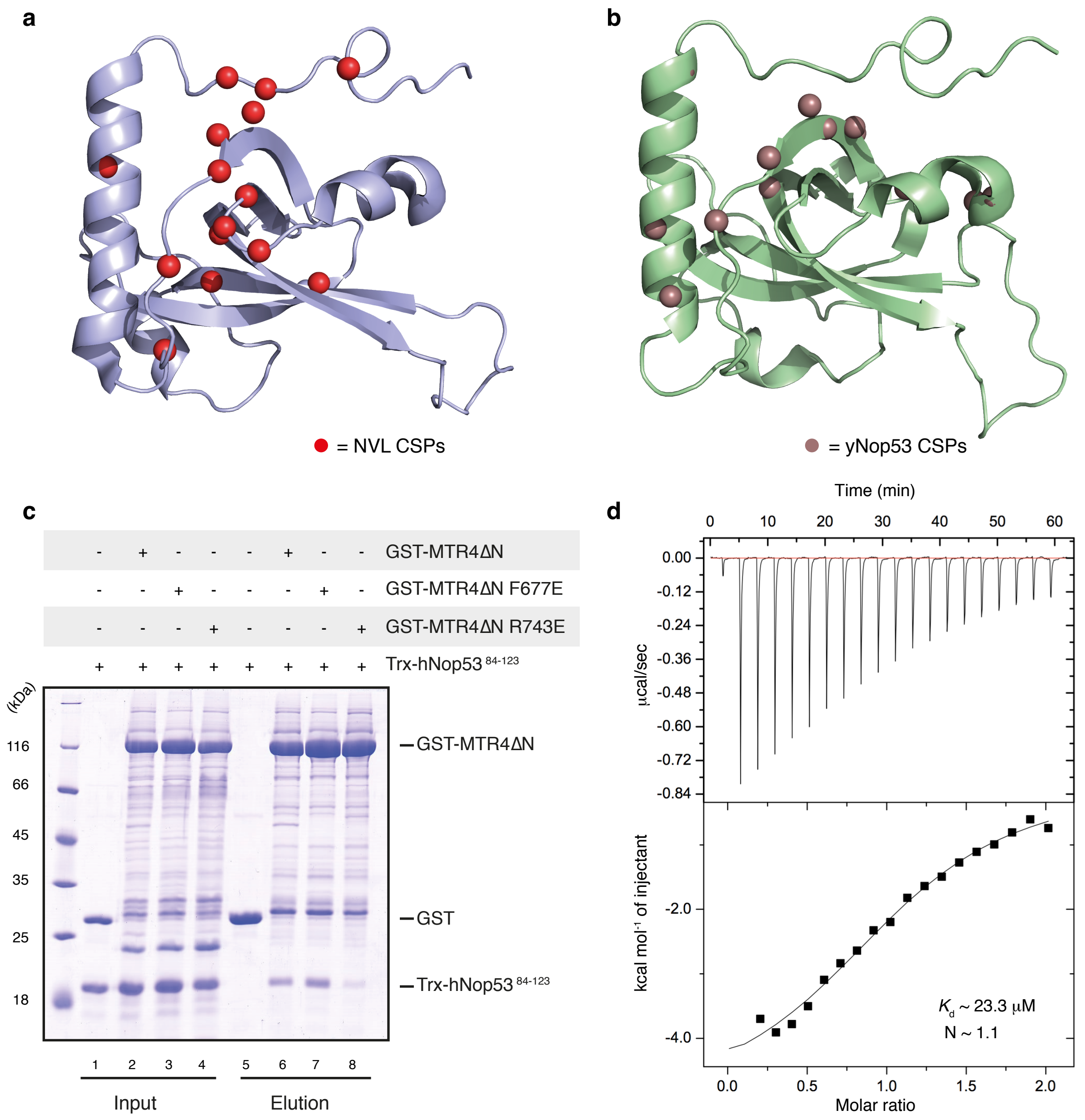

Supplementary Figure 3 (a) Structural model of MTR4 KOW (PDB 6IEH)[3] showing regions of significant backbone chemical shift perturbation (CSPs) upon NVL titration highlighted as red spheres. (b) yMtr4 KOW (PDB 5OOQ) showing significant CSPs (orange spheres) upon yNop53 titration as reported by Falk et al[4]. (c) Protein co-precipitations by pull down assays. GST tagged MTR4ΔN or the corresponding MTR4ΔN mutants were incubated with the AIM containing region of human Nop53 (Trx-hNop53 $^{84\text{-}123}$) before co-precipitation with glutathione sepharose beads. A total of 1% of the input (left) and 30% of the eluates (right) were analyzed on 15% SDS-PAGE gels and visualized by staining with coomassie brilliant blue. (d) ITC experiment of MTR4 KOW with hNop53 $^{84\text{-}123}$. The filled squares show reference corrected titration of hNop53 $^{84\text{-}123}$ into the MTR4 KOW containing cell. The number of calculated binding sites (N), and dissociation constants (K_d) are shown in the inset.

Supplementary Figure 4: Features of the NVL-MTR4 crystal structure

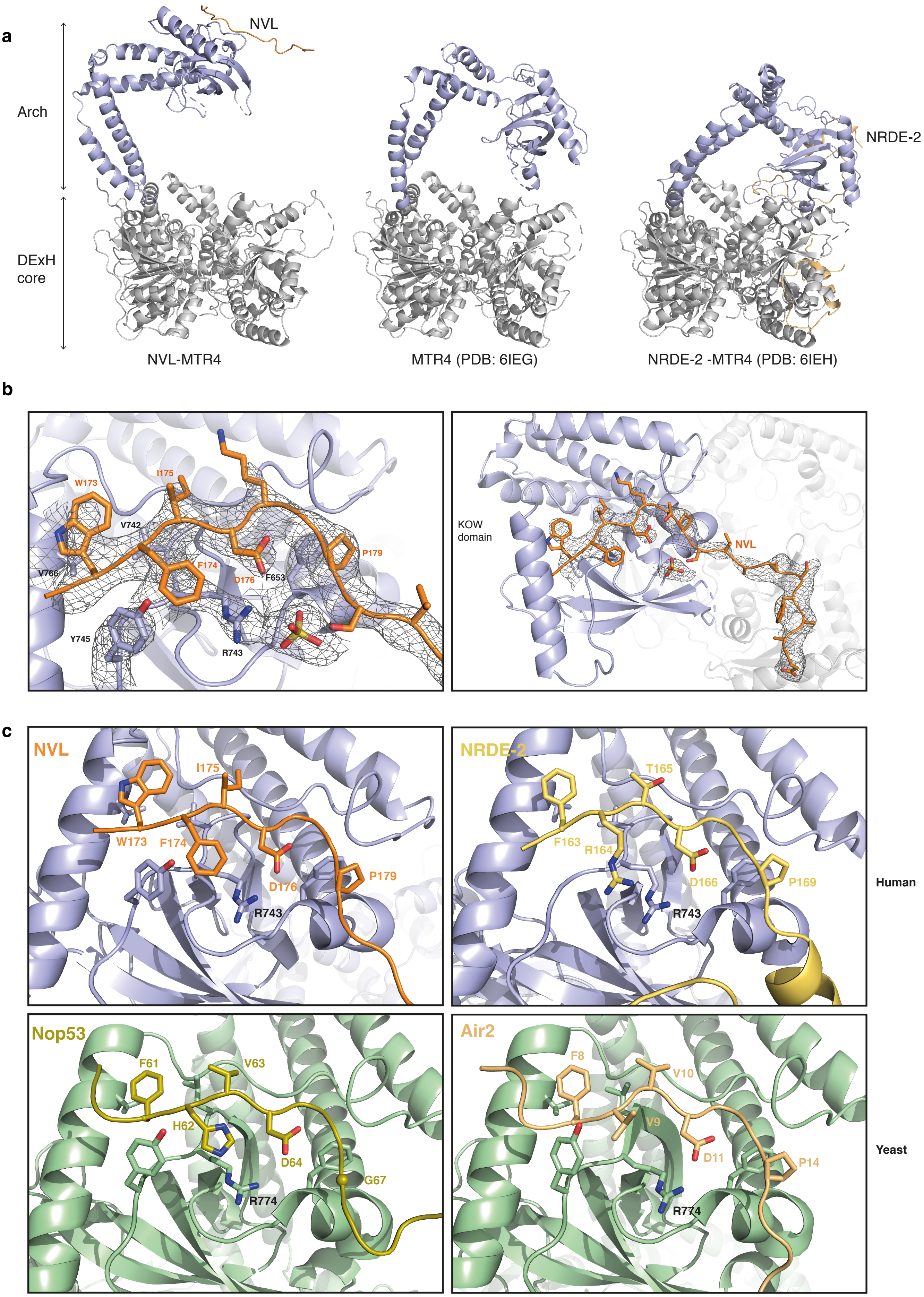

Supplementary Figure 4 (a) Comparison of the crystal structure of the NVL-MTR4 complex with crystal structure of MTR4 alone and the NRDE-2-MTR4 complex (PDB 6IEG and 6IEH)[3]. The structures were aligned based on the DExH core region (colored in grey) to represent the difference in orientation of the arch region (colored in light blue) with respect to the DExH core. (b) Zoom-in view of the interactions between MTR4 KOW domain (light blue) and NVL (orange) as displayed in Fig. 3d. The model is overlaid with the refined 2mFo-DFc map (grey mesh) showing the density for NVL and the interacting residues of MTR4. The residues of interest are labeled and the map is contoured at 1.0σ. (c) Zoom in view of MTR4-NVL crystal structure showing the ordered region of NVL (orange). The model is overlaid with the 2mFo-DFc omit map calculated in PHENIX omitting the NVL residues. The map is contoured at 1.0σ. (d) Zoom-in view of the KOW-AIM interfaces in NVL-MTR4, NRDE-2-MTR4 (PDB 6IEH[3]), Nop53-Mtr4 (PDB 5OOQ[4]) and Trf4-Air2-Mtr4 (PDB 4U4C[5]) crystal structures. KOW domains of the human MTR4 and yeast Mtr4 are colored in light blue and green respectively. NVL, NRDE-2, Nop53 and Air2 are colored in orange, yellow orange, olive and pale orange respectively and the residues of interest are labeled.

Supplementary Figure 5: Features of the NVL-MTR4 crystal structure and structure based mutagenesis

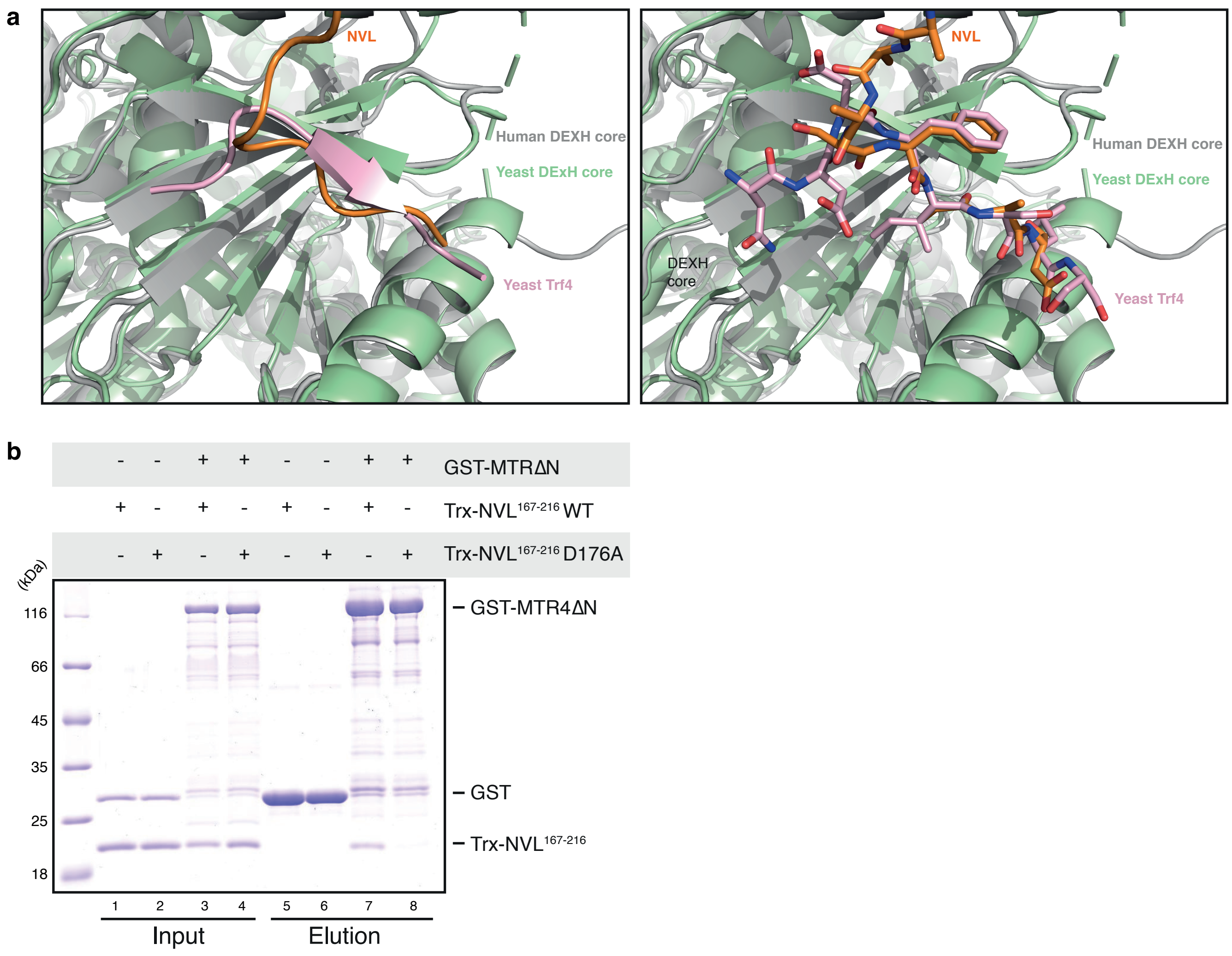

Supplementary Figure 5 (a) Superposition of DExH cores NVL-MTR4 (grey) and Trf4-Air2-Mtr4 (light green; PDB 4U4C[5]) showing that the NVL fragment (orange) from a symmetry related molecule aligns with the Trf4 fragment in Trf4-Air2-Mtr4 structure. NVL and Trf4 are shown in cartoon representation (left panel) and in stick representation (right panel) for clarity. (b) Protein co-precipitations by pull down assays. GST tagged MTR4ΔN was incubated with Trx-NVL$^{167\text{-}216}$ WT or Trx-NVL$^{167\text{-}216}$ D176A before co-precipitation with glutathione sepharose beads. A total of 1% of the input (left) and 30% of the eluates (right) were analyzed on 12% SDS-PAGE gels and visualized by staining with coomassie brilliant blue.

Supplementary Figure 6: ZCCHC8 harbors both canonical and non-canonical AIMs

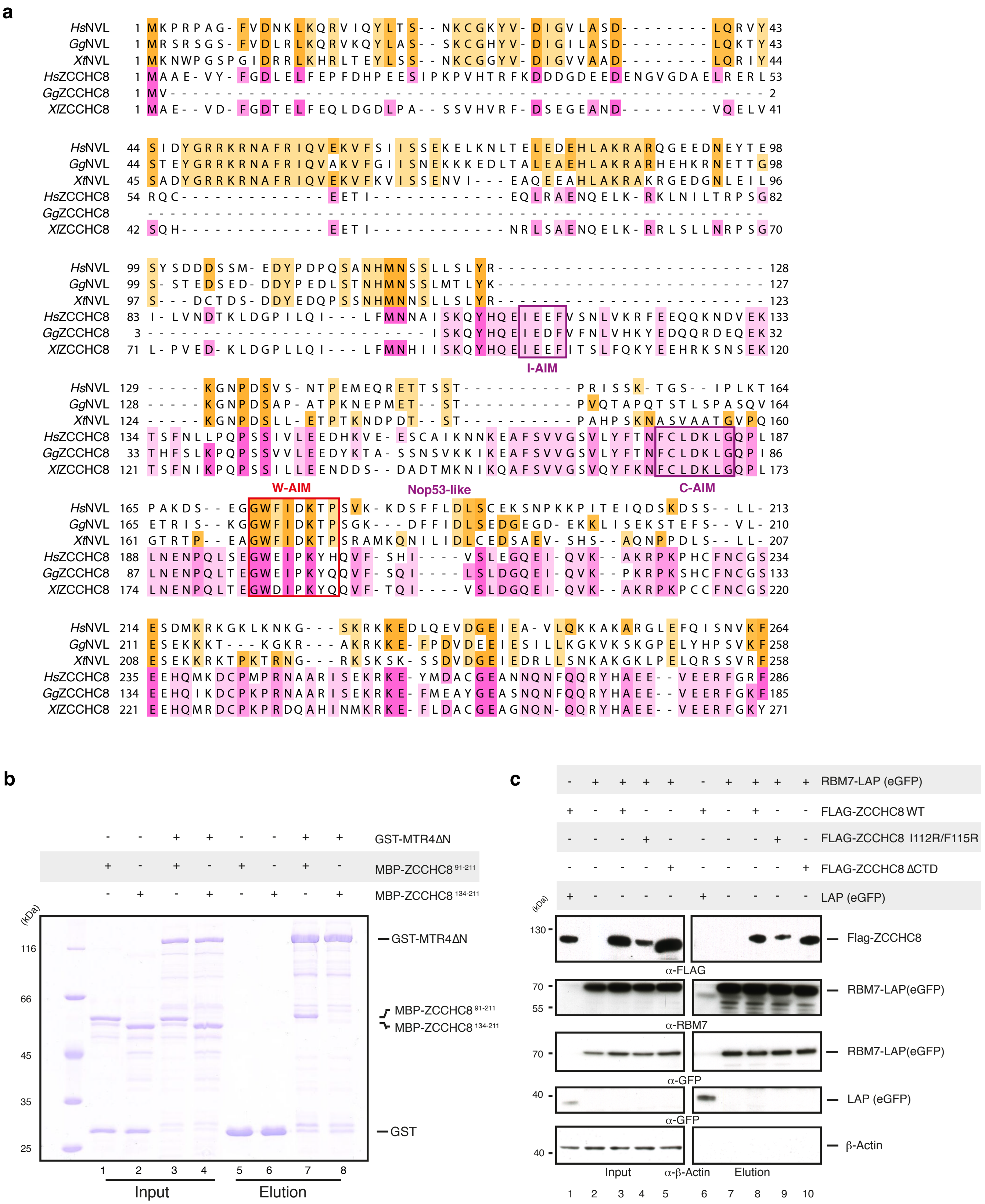

Supplementary Figure 6 (a) Sequence alignment of the N-termini of NVL (orange) and ZCCHC8 (pink), highlighting W-AIM (red box), the respective Nop53-like C-AIM (LFXϕD) and ZCCHC8 specific I-AIM (purple boxes). The sequences were obtained from the UniProt database and aligned using the T-coffee server[1]. *Hs* stands for *Homo sapiens*, Gg for *Gallus gallus*, *Xt* for *Xenopus tropicalis and, Xl* for *Xenopus laevis*. (b) Protein co-precipitations by pull down assays. GST tagged MTR4ΔN was incubated with MBP-ZCCHC8$^{91-211}$ or MBP-ZCCHC8$^{134-211}$ before co-precipitation with glutathione sepharose beads. A total of 1% of the input (left) and 30% of the eluates (right) were analyzed on 10% SDS-PAGE gels and visualized by staining with coomassie brilliant blue. (c) Cellular co-IP assay. FLAG-tagged ZCCHC8 constructs (WT, IF-mutant, CTD-deletion) were transiently expressed in cells stably expressing RBM7-LAP. After precipitation of RBM7 taking advantage of the LAP tag, a total of 0.5% of the input (left) and 8.0% of the eluates (right) were analyzed on 4-12% SDS-PAGE gel followed by western blotting. The primary antibody used is indicated below the panel.

Supplementary Figure 7: Putative ligand binding phenylalanine in tudor domains is conserved in MTR4 KOW

a

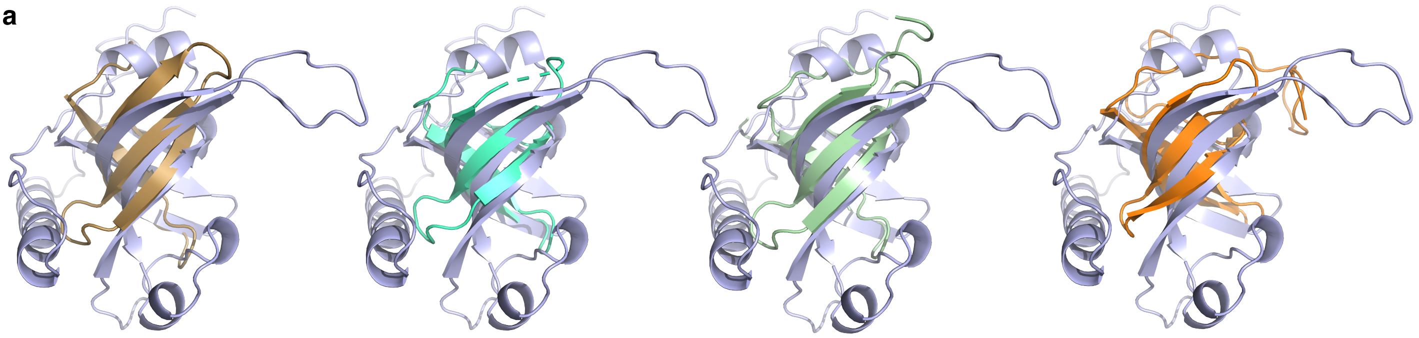

b

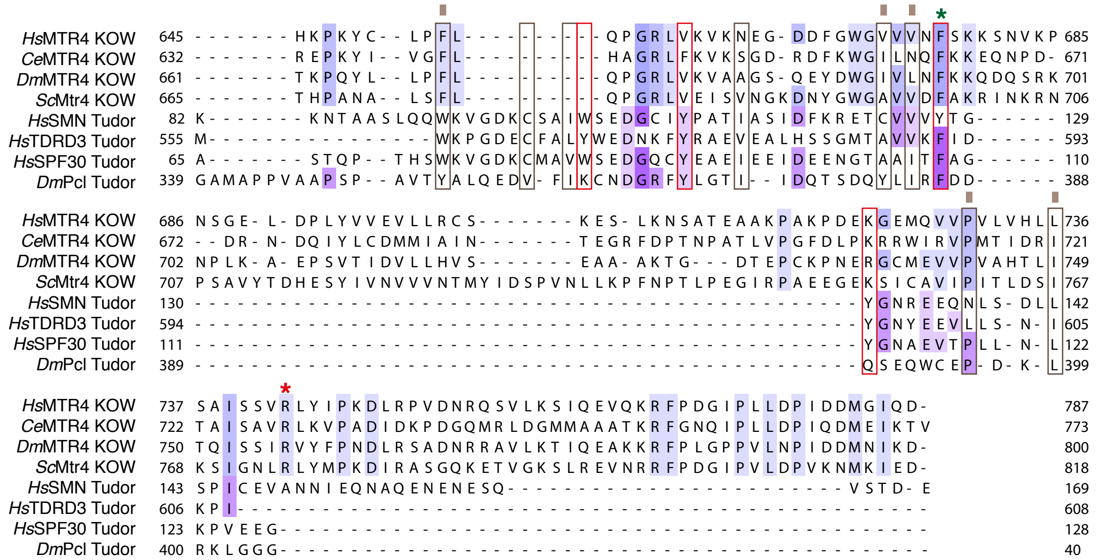

c

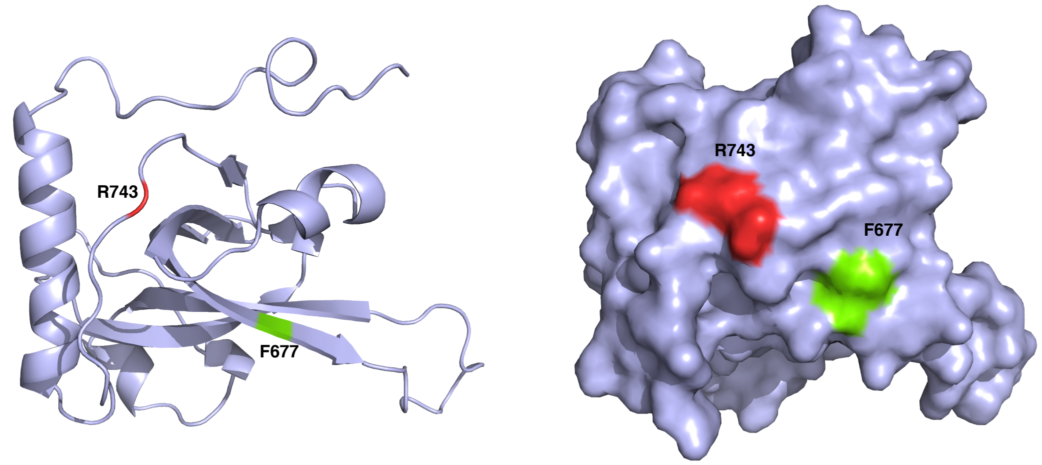

Supplementary Figure 7 (a) Structural superposition of MTR4 KOW model (light blue) (PDB 6IEH[3]) with structures of SMN tudor domain (gold) (PDB 1G5V)[6], TDRD3 tudor domain (turquoise) (PDB 3S6W)[7], SPF30 tudor domain (smudge green) (PDB 4A4F)[8] and Pcl tudor domain (orange) (PDB 2XK0)[9]. The superpositions were performed in PyMOL graphics system, version 2.2 (Schrödinger LLC). (b) Sequence alignment of representative MTR4 KOW (blue) and tudor (purple) domains, highlighting hydrophobic core residues (brown box) and putative substrate binding residues (red box) of tudor domains. The hydrophobic core resides conserved between KOW and tudor sequences are marked with brown rectangles (▪). The residues of interest in MTR4 KOW, Phe677 and Arg743, are marked by green and red asterisks respectively. The sequences were obtained from UniProt database and aligned using the T-coffee server[1]. *Hs* stands for *Homo sapiens*, *Ce* for *Caenorhabditis elegans*, *Dm* for *Drosphila melanogaster*, and *Sc* for *Saccharomyces cerevisiae*. (c) Cartoon and surface representation of MTR4 KOW (light blue) (PDB 6IEH)[3] highlighting the AIM interacting arginine (red) and putative ligand binding phenylalanine (green).

Supplementary Figure 8 : Arch interacting regions of NVL and ZCCHC8 do not influence MTR4 activity.

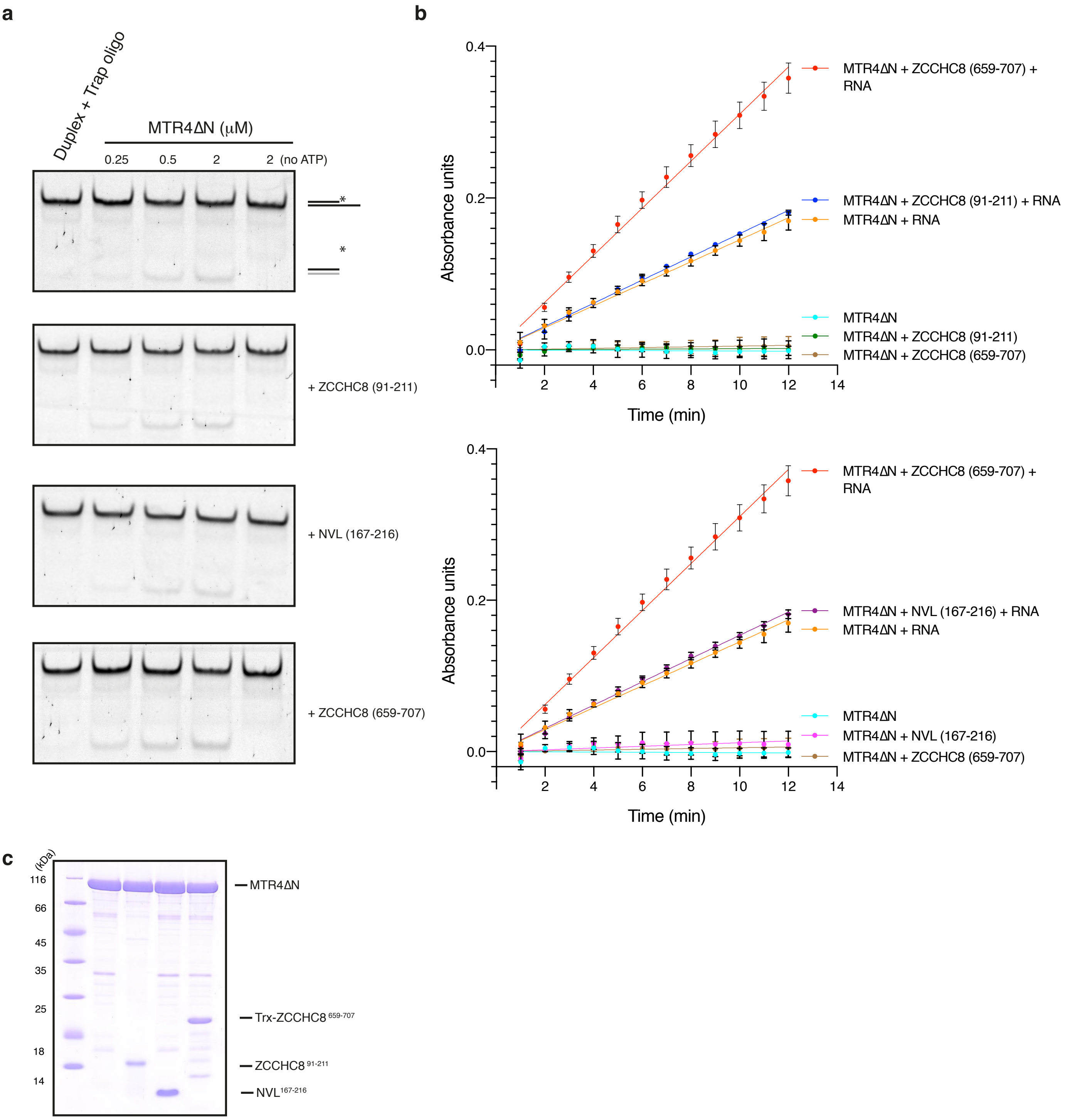

Supplementary Figure 8 (a) End point helicase activity assay of MTR4ΔN, MTR4ΔN with KOW-binding regions of NVL and ZCCHC8, and MTR4 with C-terminal domain of ZCCHC8 on RNA duplex substrates. (b) Time course of ATP hydrolysis by MTR4ΔN alone or in presence of RNA and KOW-binding regions of ZCCHC8 (upper panel) and NVL (lower panel). The data show mean (n=3) with standard deviation plotted as error bars. ZCCHC8 C-terminal domain, which is known to stimulate ATPase activity of MTR4[10], is used as positive control (c) Coomassie stained SDS-PAGE gel showing the proteins used in the helicase and ATPase assays.

Supplementary Figure 9 : Stereo view of electron density at NVL-MTR4 interface

a

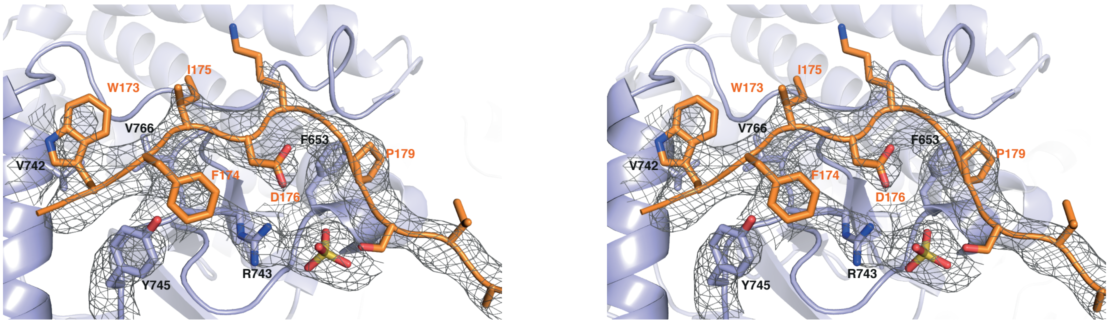

b

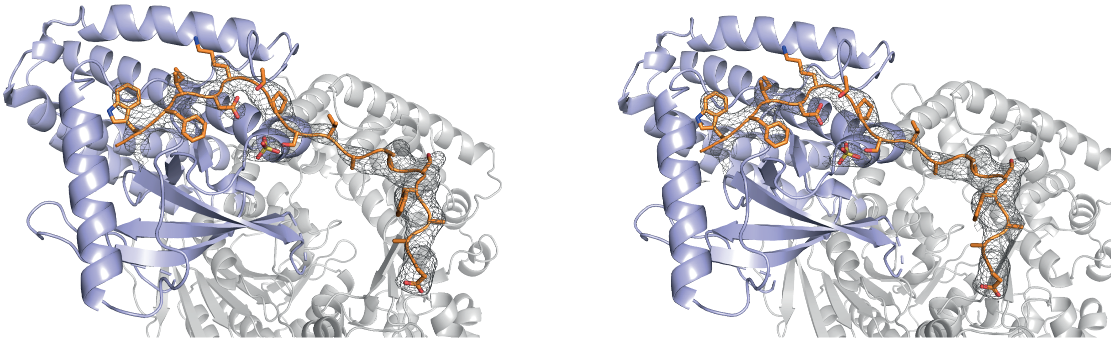

Supplementary Figure 9 (a) Stereo zoom-in view of the interactions between MTR4 KOW domain (light blue) and NVL (orange) as displayed in Fig. 3d. The model is overlaid with the refined 2mFo-DFc map (grey mesh) showing the density for NVL and the interacting residues of MTR4. The residues of interest are labeled and the map is contoured at 1.0σ. (b) Stereo zoom in view of MTR4-NVL crystal structure showing the ordered region of NVL (orange). The model is overlaid with the 2mFo-DFc omit map calculated in PHENIX omitting the NVL residues. The map is contoured at 1.0σ.

Supplementary Figure 10: Uncropped gels & blots

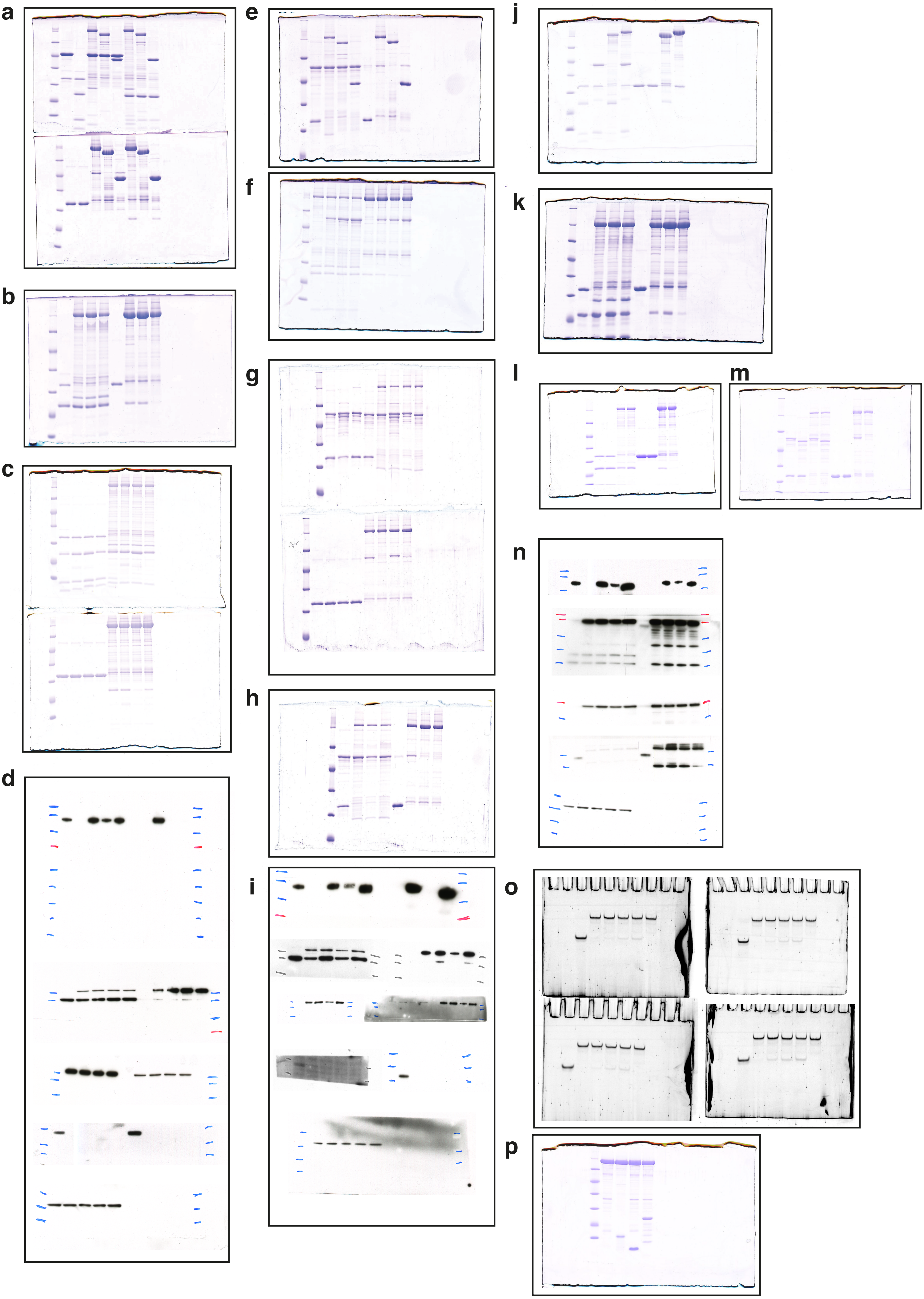

Supplementary Figure 10 (a) Fig.1b (b) Fig.2d (c) Fig.3b (d) Fig.3e (e) Fig.4b (f) Fig.4d (g) Fig.5b (h) Fig.5c (i) Fig.5d (j) Supplementary Fig.1c (k) Supplementary Fig.3c (l) Supplementary Fig.5b (m) Supplementary Fig.6b (n) Supplementary Fig.6c (o) Supplementary Fig.8a (p) Supplementary Fig.8c.

Supplementary Table 1: List of primers for generating constructs used in this study.

Primer	Sequence (5'-3')
MTRΔN-Fwd	ccaggggcccgactcgatgatttttggaaagaagcccaggatagaagagtc
MTRΔN-Rev	cagaccgccaccgactgcttacaagtagaggctggcagcaaacacaatatctctcttg
MTRΔNΔarch-Fwd	gctttttcagttccagaatgttattagctctggctcgggacaggccgttattcagctggatgacc
MTRΔNΔarch-Rev	ggtcatccagctgaataacggcctgtcccgagccagagctaataacattctggaactgaaaaaagc
MTR4 KOW-Fwd	ccaggggcccgactcgatgcacaaaccaaaatactgcttaccttttctac
MTR4 KOW-Rev	cagaccgccaccgactgcttaatcttgaatgcccatatcatcaataggg
NVL(1-266)-Fwd	ccaggggcccgactcgatgaagcccagacctgcagggttcg
NVL(1-266)-Rev	cagaccgccaccgactgcttaatcttcaaacttcacgttggagatctgg
NVL(167-216)-Fwd	ccaggggcccgactcgatgaaagattctgaaggaggatggtttattgac
NVL(167-216)-Rev	cagaccgccaccgactgcttaatcactctccaaaagagaagaatcttttgaatcc
NVL(167-216)-eYFP-cterm fusion-Fwd	tcaaaagattcttctcttttggagagtgatggcagcatggtgagcaagggcgag
NVL(167-216) eYFP cterm fusion-Rev	ctcgcccttgctcaccatgctgccatcactctccaaaagagaagaatcttttga
MTRΔN F677E-Fwd	tgactttggctggggagtagtggtgaatgagtcaaaaaagtcaaatgttaag
MTRΔN F677E-Rev	cttaacatttgacttttttgactcattcaccactactccccagccaaagtca
MTRΔN R743E-Fwd	gtctttaggaatgtaaagctcaacactgctgatagcagacaggag
MTRΔN R743E-Rev	ctcctgtctgctatcagcagtgttgagctttacattcctaaagac
MTRΔN for crystallization-Fwd	ccaggggcccgactcggaaaacctgtatttccagggaacagatgaacccatttttggaaagaagc
NVL(167-216) F186A/D189R-Fwd	gtgtaaagaaagacagtgctttcttgcgcctgtcatgtgagaaaagtaatcc
NVL(167-216) F186A/D189R-Rev	ggattacttttctcacatgacaggcgcaagaaagcactgtctttctttacac
NVL(167-216) W173A-Fwd	ccaggggcccgactcgaaagattctgaaggaggagcgtttattgac
NVL(167-216) I175E-Fwd	ccaggggcccgactcgaaagattctgaaggaggatggtttgaagac
NVL FL-Fwd	taagcagatatcatgaagcccagacctgcag
NVL FL-Rev	tgcttagcggccgcccggctgagggactcct
NVL FL W173A/I175E-Fwd	ctgccaaagattctgaaggaggagcgtttgcggacaaaaccccaagtgtaaag
NVL FL W173A/I175E-Rev	ctttacacttggggttttgtccgcaaacgctcctccttcagaatctttggcag
NVL FL ΔW-AIM-Fwd	ccattcccttgaagacccctgccaaagattctgaaggaagtgtaaagaaagacagtttttcttggacctg
NVL FL ΔW-AIM-Rev	caggtccaagaaaaactgtctttctttacacttccttcagaatctttggcaggggtcttcaagggaatgg
ZCCHC8(91-211)-Fwd	aagttctgttccaggggcccatggatggacctatattacagattctattcatgaacaatg
ZCCHC8(91-211)-Rev	ccccagaacatcaggttaatggcgttaaacaatgtggctgaagacttgatggtacttg
ZCCHC8(91-211)-eYFP cterm fusion-Fwd	catcaagtcttcagccacattgttggcagcatggtgagcaagggcgag
ZCCHC8(91-211)-eYFP cterm fusion-Rev	ctcgcccttgctcaccatgctgccaacaatgtggctgaagacttgatg
ZCCHC8(91-211) F178A/D181R-Fwd	gaagcggttgccccaatttacgaaggcaagcattagtaaaatacaggacacttcctacaaca
ZCCHC8(91-211) F178A/D181R-Rev	tgttgtaggaagtgtcctgtattttactaatgcttgccttcgtaaattggggcaaccgcttc
ZCCHC8(91-211) W198A/K202E-Rev	gcaaagcaccgtcgttaaacaatgtggctgaagacttgatggtactcgggtatttcagctccttcggaaagctg
ZCCHC8(91-211)/FL I112R/F115R-Fwd	ttcatgaacaatgctatttcaaagcaatatcatcaagaaagagaggaacgtgtatcaaatttagtaaaaagatttgag
ZCCHC8(91-211)/FL I112R/F115R-Rev	ctcaaatctttttactaaatttgatacacgttcctctctttcttgatgatattgctttgaaatagcattgttcatgaa
ZCCHC8 FL-Fwd	taagcagatatcatggccgcagaggtgtattt
ZCCHC8 FL-Rev	tgcttagcggccgcttcagaggccttttttgtttttctg

yMtr4-Fwd	ccaggggcccgactcgatggattctactgatctgttcgatgttttcgagg
yMtr4-Rev	cagaccgccaccgactgcttataaatacaaagaaccagcagatacgatatctctatg
Rix7(1-206)-Fwd	ccagggagcagcctcgatggttaaagtaaagtcgaaaaagaactcatt
Rix7(1-206)-Rev	gcaaagcaccggcctcgttaggatttcagagacgaattaggtggagatc
hNop53(84-123)-Fwd	ccaggggcccgactcgatggaaaaactcttcttcgtggacactg
hNop53(84-123)-Rev	cagaccgccaccgactgcttattctcgaggatgaggtcaacc
NVL D176A-Fwd	ccaggggcccgactcgatgaaagattctgaaggaggatggtttattgccaaaac
ZCCHC8(134-211)-Fwd	aagttctgttccaggggcccatgacttcctttaatcttttgccccagc
ZCCHC8 ΔCTD-Rev	tgcttagcggccgcgctatgaattttagtggccgttg
ZCCHC8 (659-707)-Fwd	ccaggggcccgactcgatgcctatacctgacatgagcaaatttgcaac
ZCCHC8 (659-707)-Rev	cagaccgccaccgactgcttattattcagaggcctttttgtttttctgc

*All primers contain overhangs to facilitate ligation independent cloning

Supplementary References

1. Di Tommaso, P. et al. T-Coffee: a web server for the multiple sequence alignment of protein and RNA sequences using structural information and homology extension. *Nucleic Acids Res* **39**, W13-7 (2011).
2. Oates, M.E. et al. D(2)P(2): database of disordered protein predictions. *Nucleic Acids Res* **41**, D508-16 (2013).
3. Wang, J. et al. NRDE2 negatively regulates exosome functions by inhibiting MTR4 recruitment and exosome interaction. *Genes Dev* (2019).
4. Falk, S. et al. Structural insights into the interaction of the nuclear exosome helicase Mtr4 with the preribosomal protein Nop53. *RNA* **23**, 1780-1787 (2017).
5. Falk, S. et al. The molecular architecture of the TRAMP complex reveals the organization and interplay of its two catalytic activities. *Mol Cell* **55**, 856-867 (2014).
6. Selenko, P. et al. SMN tudor domain structure and its interaction with the Sm proteins. *Nat Struct Biol* **8**, 27-31 (2001).
7. Liu, K. et al. Crystal structure of TDRD3 and methyl-arginine binding characterization of TDRD3, SMN and SPF30. *PLoS One* **7**, e30375 (2012).
8. Tripsianes, K. et al. Structural basis for dimethylarginine recognition by the Tudor domains of human SMN and SPF30 proteins. *Nat Struct Mol Biol* **18**, 1414-20 (2011).
9. Friberg, A., Oddone, A., Klymenko, T., Muller, J. & Sattler, M. Structure of an atypical Tudor domain in the Drosophila Polycomblike protein. *Protein Sci* **19**, 1906-16 (2010).
10. Puno, M.R. & Lima, C.D. Structural basis for MTR4-ZCCHC8 interactions that stimulate the MTR4 helicase in the nuclear exosome-targeting complex. *Proc Natl Acad Sci U S A* **115**, E5506-E5515 (2018).
