# Supplementary material for: The MTR4 helicase recruits nuclear adaptors of the human RNA exosome using distinct arch-interacting motifs
Source: Nat Commun. 2019 Jul 29;10:3393. doi: 10.1038/s41467-019-11339-x (PMC6662825; doi:10.1038/s41467-019-11339-x)
Supplement: Supplementary file 3 — Reporting Summary [file 41467_2019_11339_MOESM3_ESM.pdf]

nature research

Corresponding author(s): Elena Conti and Sebastian Falk

Last updated by author(s): Jun 20, 2019

Reporting Summary

Nature Research wishes to improve the reproducibility of the work that we publish. This form provides structure for consistency and transparency in reporting. For further information on Nature Research policies, see Authors & Referees and the Editorial Policy Checklist.

Statistics

For all statistical analyses, confirm that the following items are present in the figure legend, table legend, main text, or Methods section.

n/a	Confirmed	
☐	☒	The exact sample size (*n*) for each experimental group/condition, given as a discrete number and unit of measurement
☐	☒	A statement on whether measurements were taken from distinct samples or whether the same sample was measured repeatedly
☒	☐	The statistical test(s) used AND whether they are one- or two-sided<br>*Only common tests should be described solely by name; describe more complex techniques in the Methods section.*
☒	☐	A description of all covariates tested
☒	☐	A description of any assumptions or corrections, such as tests of normality and adjustment for multiple comparisons
☐	☒	A full description of the statistical parameters including central tendency (e.g. means) or other basic estimates (e.g. regression coefficient) AND variation (e.g. standard deviation) or associated estimates of uncertainty (e.g. confidence intervals)
☒	☐	For null hypothesis testing, the test statistic (e.g. F, t, r) with confidence intervals, effect sizes, degrees of freedom and P value noted<br>*Give P values as exact values whenever suitable.*
☒	☐	For Bayesian analysis, information on the choice of priors and Markov chain Monte Carlo settings
☒	☐	For hierarchical and complex designs, identification of the appropriate level for tests and full reporting of outcomes
☒	☐	Estimates of effect sizes (e.g. Cohen's *d*, Pearson's *r*), indicating how they were calculated

Our web collection on statistics for biologists contains articles on many of the points above.

Software and code

Policy information about availability of computer code

Data collection	Microcal ITC200-Origin software for ITC, MO software (NanoTemper) for MST, Topspin 3.5 for NMR, DA+ at SLS for X-ray data
Data analysis	Origin 5.0 for ITC data analysis; MO software for analysis MST data (Nanotemper); CCPNMR Analysis 2.4, Sparky (http://www.cgl.ucsf.edu/home/sparky) for NMR; Xia2/DIALS in CCP4i2, Phaser, PHENIX, COOT, pyMOL for crystallographic data

For manuscripts utilizing custom algorithms or software that are central to the research but not yet described in published literature, software must be made available to editors/reviewers. We strongly encourage code deposition in a community repository (e.g. GitHub). See the Nature Research guidelines for submitting code & software for further information.

Data

Policy information about availability of data

All manuscripts must include a data availability statement. This statement should provide the following information, where applicable:

- Accession codes, unique identifiers, or web links for publicly available datasets
- A list of figures that have associated raw data
- A description of any restrictions on data availability

NMR backbone chemical shifts of the human MTR4 KOW domain were deposited at the BMRB under accession number 27831. The coordinates and the structure factors have been deposited in the Protein Data Bank with accession code PDB ID 6RO1. Other data are available from the corresponding authors upon reasonable request.

Field-specific reporting

Please select the one below that is the best fit for your research. If you are not sure, read the appropriate sections before making your selection.

☒ Life sciences ☐ Behavioural & social sciences ☐ Ecological, evolutionary & environmental sciences

For a reference copy of the document with all sections, see nature.com/documents/nr-reporting-summary-flat.pdf

Life sciences study design

All studies must disclose on these points even when the disclosure is negative.

Sample size	All experiments were performed atleast twice. MST and ATPase assays were performed in triplicate.
Data exclusions	In the ITC measurements the first data point is excluded from the binding curve analysis as this is common practice.<br>Crystallographic data were cutoff based on the CC1/2 parameter.
Replication	The MST and ATPase experiments were performed in triplicates and the error bars shown correspond to the calculated standard deviation.
Randomization	No experiments that require randomization were performed.
Blinding	No experiments that require blinding were performed.

Reporting for specific materials, systems and methods

We require information from authors about some types of materials, experimental systems and methods used in many studies. Here, indicate whether each material, system or method listed is relevant to your study. If you are not sure if a list item applies to your research, read the appropriate section before selecting a response.

Materials & experimental systems

n/a	Involved in the study	
☐	☒	Antibodies
☐	☒	Eukaryotic cell lines
☒	☐	Palaeontology
☒	☐	Animals and other organisms
☒	☐	Human research participants
☒	☐	Clinical data

Methods

n/a	Involved in the study	
☒	☐	ChIP-seq
☒	☐	Flow cytometry
☒	☐	MRI-based neuroimaging

Antibodies

Antibodies used	anti-FLAG: supplier: Sigma-aldrich, cat: F1804, clone: M2, lot: SLBF6631. Dilution: 1:50 000<br>anti-GFP: supplier: SCBT, cat: sc-9996, clone: B2 lot: F1115. Dilution: 1:1000<br>anti-MTR4: supplier: Abcam, cat: ab70551, lot: GR3205980-1. Dilution: 1:4000<br>anti-B-actin: supplier: Sigma-aldrich. cat: a2228, clone: AC-74. Dilution: 1:100 000<br>anti-RBM7: supplier: Human Protein Atlas. cat: HPA013993, lot: D116570. Dilution: 1:1000
Validation	FLAG: Validated by immunoprecipitation and western blot in the present manuscript. Validated by overexpression and immunofluorescence (data on manufacturer's website).<br>GFP: Validated by immunoprecipitation and western blot in the present manuscript. Validated by overexpression western blot (data on manufacturer's website)<br>MTR4: Validated by immunoprecipitation and western blot in the present manuscript. Validated by western blot (data on manufacturer's website).<br>B-actin: Validated by western blot and immunofluorescence (data on manufacturer's website).<br>RBM7: Validated by immunoprecipitation and western blot in the present manuscript. Validated by immunofluorescence and western blot (data on manufacturer's website).

Eukaryotic cell lines

Policy information about cell lines

Cell line source(s)	HeLa Kyoto LAP, HeLa Kyoto MTR4-LAP and HeLa Kyoto RBM7-LAP<br>Poser, Ina et al. “BAC TransgeneOmics: a high-throughput method for exploration of protein function in mammals” Nature methods vol. 5,5 (2008): 409-15.

Authentication	Overexpression of LAP, MTR4-LAP, and RBM7-LAP were authenticated by immunoprecipitation and western blot using GFP, MTR4, and RBM7 antibodies. No further authentication of HeLa cell lines was performed.
Mycoplasma contamination	Cells were tested negative for mycoplasma contamination.
Commonly misidentified lines (See ICLAC register)	None.
