# Supplementary material for: The MTR4 helicase recruits nuclear adaptors of the human RNA exosome using distinct arch-interacting motifs
Source: Nat Commun. 2019 Jul 29;10:3393. doi: 10.1038/s41467-019-11339-x (PMC6662825; doi:10.1038/s41467-019-11339-x)
Supplement: Supplementary file 2 — Peer Review [file 41467_2019_11339_MOESM2_ESM.pdf]

Reviewers' comments:

Reviewer #1 (Remarks to the Author):

The authors report an analysis of the interactions of cofactors with the helicase MTR4. The work is technically excellent and the results will be of considerable value to the fields of RNA processing and surveillance. The obvious weakness is the lack of functional validation of the significance of the reported interactions. However, the results will no doubt form the basis of future functional analyses, very likely by the authors. I am happy to recommend publication in principal, and have only minor comments.

Minor comments:

1) The MS is not always easy to read. Transitions between experiments and explanations of the links could be improved. It is not always clear why sites were selected or the logic behind this, even when the final results are successful. The need to refer back to previous figures is unhelpful.

For example; It was not really clear why the NVL fragment from 167 to 216 was selected? Why did they decide to mutate at the so-called I-AIM in ZCCHC8, since it does not clearly resemble any other known AIM? Did they test several residues for mutation in ZCCHC8 to impair binding to Mtr4 and only Phe677 worked?

2) More discussion would be helpful: Points that might be elaborated include why some motifs that closely match AIM (such as the W-AIM in ZCCHC8) do not appear to be involved in association under the conditions used? Could they be involved in different conditions in vivo ?

Reviewer #2 (Remarks to the Author):

MTR4 is a helicase that functions with the RNA exosome to promote processing and decay. It remodels exosome substrates using its RNA dependent ATPase activity whereas adaptor protein target MTR4 to different substrates.

The manuscript by Lingaru et al reports distinct short-linear interaction motifs (SLiMS) present in the adaptor proteins, ZCCHC8 and NVL, that target human MTR4 and the exosome for ribosome processing and small nuclear RNA decay respectively. Site-directed mutagenesis, pull-down assays, and NMR chemical shift mapping indicate the newly identified motifs interact with the ARCH domain of MTR4, specifically the KOW subdomain, in a mutually exclusive manner. The newly identified arch-interacting motifs (AIMS) are dubbed W-AIM, C-AIM , and I-AIM based on signature hydrophobic amino acids important for binding. Consistent with the idea that SLiMS form linear epitopes that are not constrained by 3D structure, the newly identified motifs are not conserved in yeast adaptors for MTR4. Therefore, this story adds to a growing body of literature showing how short linear interactions motifs rapidly evolve rewire important regulatory protein complexes involved in RNA processing and decay.

The studies are well-controlled, and the interactions that are dissected in vitro are followed up by co-IP experiments carried out using proteins expressed in human cell lines (HeLa).

Overall, the manuscript is solid but somewhat light on functional studies and analyses. It could be suitable for publication in Nature Communications after the following points are addressed.

1-How do the newly discovered motifs effect MTR4 activities? Helicase and ATPase activities of in vitro reconstituted complexes could be informative.

2-When did the W-AIM motifs diverge from the yeast motifs? Are they conserved in all metazoan NVL and ZCCH4 orthologs, or specific to vertebrates, on specific to primates only?

Reviewer #3 (Remarks to the Author):

In this manuscript, Lingaraju et al. provide new insights into the regulation of the nuclear exosome by the adaptor proteins associated with the RNA helicase Mtr4 using complementary structural (NMR) and biochemical approaches. The study provides a compelling evidence that arch domain of Mtr4 engages in interaction with functionally different adaptor proteins in mutually exclusive manner – ribosome processing adaptor NVL and N-terminal part of ZCCHC8 protein that is a part of NEXT (the nuclear exosome targeting complex) complex, previously identified by Torben Jensen's lab. They characterise the interaction and identify novel motifs that are responsible for binding to Mtr4 arch domain. The study fills a gap in our understanding of the exosome regulation and furthers recently published work from Chris Lima's Lab demonstrating that C-terminal part of ZCCHC8 is important for activation of the helicase activity of the enzyme. They propose a model where different adaptor complexes are recruited to the Mtr4/exosome via interaction between conserved motifs within adaptor proteins and Mtr4 arch domain, whereas interactions between Mtr4 and adaptor proteins outside of the Mtr4 arch domain are important for activation of Mtr4.
Overall, the study advances our understanding of the fundamental process involved in regulation of RNA maturation and stability.
Comments:
1. To further support author's claim that interaction between ZCCHC8 I-AIM and Mtr4 arch is important for NEXT assembly, it would be good to check whether RBM7 co-IPs with I112R/F115R mutant

2. The study would benefit from further investigation of the impact of the interaction with the arch and the helicase activity. Helicase activity should be assessed for I112R/F115R, delta C-terminus ZCCHC8 mutants and Mtr4 delta arch.

3. Page 4, lane 2-correct …small nuclear and nuclear… to … small nuclear and nucleolar…

We thank the Reviewers for their positive comments and their constructive criticisms. We have addressed the specific criticisms raised as outlined below.

Reviewer #1 (Remarks to the Author):

Minor comments:

1) The MS is not always easy to read. Transitions between experiments and explanations of the links could be improved. It is not always clear why sites were selected or the logic behind this, even when the final results are successful. The need to refer back to previous figures is unhelpful.

In the revision, we simplified the text to better explain the links. I particular, we modified the layout of Fig 1, Fig 4 to avoid referring back to previous figures. We also reordered Fig 3, 5 arranging the panels in line with in-text referencing. There is only one instance where we could not avoid referring back to previous figures.

For example; It was not really clear why the NVL fragment from 167 to 216 was selected?

We selected this fragment based on bioinformatics analyses. The alignment of NVL sequences from different species (new Supplementary Fig.1a) shows that the evolutionary conservation in the chordate-specific insertion maps in the 167-216 region, and we therefore focused on this region.

Why did they decide to mutate at the so-called I-AIM in ZCCHC8, since it does not clearly resemble any other known AIM?

We now detail how we identified all AIM motifs we describe in this study (W-AIM, C-AIM and I-AIM). In the case of ZCCHC8, it was clear that the classical hotspot (occupied by the C-AIM) motif could not alone be responsible for the high affinity of the interaction and therefore another motif might exist that would interact with another surface (and thus not resemble other AIM motifs). We hypothesized that the second motif would be located on the N-terminal region of the 91-211 because N-terminal truncations of this region resulted in a near loss of MTR4 binding, as shown in the pull down experiment in new Supplementary Fig 6b.

Did they test several residues for mutation in ZCCHC8 to impair binding to Mtr4 and only Phe677 worked?

We made a specific hypothesis on the involvement of Phe677. The KOW domain of MTR4 is structurally similar to Tudor domains (Supplementary Fig. 7a). Tudor domains generally bind substrates at the so-called aromatic cage, a hydrophobic

pocket present of the surface of these domains. The KOW domain of MTR4 presents a hydrophobic pocket at the corresponding position (Supplementary Fig.7b, with hydrophobic residues indicated with brown boxes above the sequences). Within these residues, Phe677 and Val660 show particular conservation. In addition, Phe677 is not far from Arg743, a residue that also participates in ZCCHC8 binding. These observations pointed to a possible role of this MTR4 pocket for substrate binding and we specifically tested this hypothesis by mutating Phe677. We did not make additional mutations.

2) More discussion would be helpful: Points that might be elaborated include why some motifs that closely match AIM (such as the W-AIM in ZCCHC8) do not appear to be involved in association under the conditions used? Could they be involved in different conditions in vivo ?

In the revised version, we could determine the crystal structure of the NVL W-AIM bound to MTR4. Putting all the information together, we could define the consensus sequence of bona-fide AIMs. With insight, sequences in both NVL and ZCCHC8 that at first glance appeared to resemble known AIMs instead contain individual amino acids that are likely to prevent MTR4 binding by electrostatic repulsion. There is no reason to believe that sequences that do not function as bona-fide AIM motifs in vitro would do so in vivo. However, these sequences may well have other functions.

Reviewer #2 (Remarks to the Author):

1-How do the newly discovered motifs effect MTR4 activities? Helicase and ATPase activities of in vitro reconstituted complexes could be informative.

We performed ATPase and helicase assays, using a similar set experimental set up published by the Lima’s lab (Puno and Lima, 2018), where the authors had shown that showing that the C-terminal domain (CTD) of ZCCHC8 modulates the activity of MTR4. We find that the motifs discussed in the manuscript do not affect the activity of MTR4 in these assays (revised Supplementary Fig. 8). These results together with co-IP data (Fig.5c) suggest a division of labour in the ZCCHC8 molecule for MTR4 binding (AIMs) and for regulation (CTD).

2-When did the W-AIM motifs diverge from the yeast motifs? Are they conserved in all metazoan NVL and ZCCH4 orthologs, or specific to vertebrates, on specific to primates only?

The insertion region seems to appear for the first time in arthropods. AIM-like motifs are present only in notochordates and chordates (Supplementary Fig.1a).

Reviewer #3 (Remarks to the Author):

1. To further support author's claim that interaction between ZCCHC8 I-AIM and Mtr4 arch is important for NEXT assembly, it would be good to check whether RBM7 co-IPs with I112R/F115R mutant

We carried out the experiment suggested. The co-IP experiment (revised Supplementary Fig.6c) shows that ZCCHC8 (I112R/F115R) precipitates RBM7, in line with the notion that ZCCHC8 is the scaffold protein that can bridge the interaction between MTR4 and RBM7.

2. The study would benefit from further investigation of the impact of the interaction with the arch and the helicase activity. Helicase activity should be assessed for I112R/F115R, delta C-terminus ZCCHC8 mutants and Mtr4 delta arch.

We performed ATPase and helicase assays (see answer to Reviewer 2).

3. Page 4, lane 2-correct …small nuclear and nuclear… to … small nuclear and nucleolar…
Corrected.

REVIEWERS' COMMENTS:

Reviewer #1 (Remarks to the Author):

The revised MS has addressed the points raised in my initial review, and has clearly been improved. I am happy to support publication.

Reviewer #2 (Remarks to the Author):

The authors have addressed my concerns; the manuscript is now suitable for publication.

Reviewer #3 (Remarks to the Author):

Authors have fully addressed all the points raised by the reviewers. I am happy to recommend this manuscript for publication
